# Acupuncture for post-stroke depression: a systematic review and meta-analysis

**DOI:** 10.1186/s12906-021-03277-3

**Published:** 2021-04-01

**Authors:** Ran Liu, Kun Zhang, Qiu-yu Tong, Guang-wei Cui, Wen Ma, Wei-dong Shen

**Affiliations:** 1 Department of Acupuncture, Shuguang Hospital, Shanghai University of Traditional Chinese Medicine, Shanghai, 201203 China; 2 Institute of Acupuncture and Anesthesia, Shuguang Hospital, Shanghai University of Traditional Chinese Medicine, Shanghai, 201203 China

**Keywords:** Acupuncture, Post-stroke depression, Systematic review, Meta-analysis, Effectiveness, Safety

## Abstract

**Background:**

Acupuncture for post-stroke depression (PSD) has been evolving, but uncertainty remains. To assess the existing evidence from randomized clinical trials (RCTs) of acupuncture for PSD, we sought to draw conclusions by synthesizing RCTs.

**Methods:**

An exhaustive literature search was conducted in seven electronic databases from their inception dates to April 19, 2020, to identify systematic reviews (SRs) and meta-analyses (MAs) on this topic. The primary RCTs included in the SRs/MAs were identified. We also conducted a supplementary search for RCTs published from January 1, 2015, to May 12, 2020. Two reviewers extracted data separately and pooled data using RevMan 5.3 software. The quality of evidence was critically appraised with the Grades of Recommendation, Assessment, Development and Evaluation (GRADE) system.

**Results:**

A total of 17 RCTs involving 1402 patients were included. Meta-analysis showed that participants who received a combination of acupuncture and conventional treatments exhibited significantly lower scores on the HAM-D_17_, HAM-D_24_ and HAM-D (MD, − 5.08 [95% CI, − 6.48 to − 3.67], *I*^2^ = 0%), (MD, − 9.72 [95% CI, − 14.54 to − 4.91], *I*^2^ = 65%) and (MD, − 2.72 [95% CI, − 3.61 to − 1.82], respectively) than those who received conventional treatment. However, there was no significant difference in acupuncture versus antidepressants in terms of the 17-item, 24-item and HAM-D scales (MD, − 0.43 [95% CI, − 1.61 to 0.75], *I*^2^ = 51%), (MD, − 3.09 [95% CI, − 10.81 to 4.63], *I*^2^ = 90%) and (MD, − 1.55 [95% CI, − 4.36 to 1.26], *I*^2^ = 95%, respectively). For adverse events, acupuncture was associated with fewer adverse events than antidepressants (RR, 0.16 [95% CI, 0.07 to 0.39], *I*^2^ = 35%), but there was no significant difference in the occurrence of adverse events between the combination of acupuncture and conventional treatments versus conventional treatments (RR, 0.63 [95% CI, 0.21 to 1.83], *I*^2^ = 38%). The quality of evidence was low to very low due to the substantial heterogeneity among the included studies.

**Conclusions:**

The current review indicates that acupuncture has greater effect on PSD and better safety profile than antidepressants, but high-quality evidence evaluating acupuncture for PSD is still needed.

**Supplementary Information:**

The online version contains supplementary material available at 10.1186/s12906-021-03277-3.

## Background

Stroke is the second largest cause of mortality and disability in the world according to the Global Burden of Diseases, Injuries, and Risk Factors Study (GBD), contributing to a high burden of disease [1]. Approximately 31.1% of stroke survivors will develop post-stroke depression (PSD), which is characterized by long-term persistent depression, often complicating the course of stroke and adversely affecting functional recovery and quality of life [2–6]. In addition, PSD could also raise the risk of mortality in stroke survivors and threaten the life security of these patients [7]. Currently, antidepressants, primarily 5-hydroxytryptamine (5-HT) reuptake inhibitors (SSRIs), are used clinically to prevent and treat PSD, but the risk of cerebral haemorrhage and recurrent stroke associated with this approach cannot be ignored [8, 9]. Therefore, there is an urgent need for innovative strategies including more effective and secure intervention in PSD [10].

Acupuncture is widely used in the treatment of PSD, and the number of studies on acupuncture for PSD has been recently increasing. Although more than 5 systematic reviews (SRs) and meta-analyses (MAs) [11–15] were conducted to appraisal the effect of acupuncture on PSD, none arrived at a consistent conclusion. The reason for that is the wide variations in interventions, qualities, and outcomes selected in these studies. For example, some of these SRs and MAs included randomized controlled trials (RCTs) with acupuncture, acupressure, acupoint injection or moxibustion, which could increase the risk of heterogeneity. In addition, some SRs/MAs failed to report outcomes according to different criteria, such as different versions of the Hamilton Rating Scale for Depression (HAM-D) or different definitions of effective rate and curative rate, which might cause estimate instability during the evaluation process. In addition, more recent trials about acupuncture for PSD have been published and add to the evidence of the association between acupuncture and the degree of depression. A trial conducted in 2018 revealed that patients with moderate or severe depression who received a combination of acupuncture and medication experienced visible depression relief [16]. This reduction in depression was also certified in a 2019 study [17].

In light of the growing number of studies of acupuncture for PSD and the ensuing need for critical evaluation, we conducted a new MA and used the GRADE system to assess the evidence quality for the outcomes of interest to discover the true efficacy and gaps in this field and to provide recommendations for clinical practice.

## Methods

This MA was performed according to the Cochrane Handbook for Systematic Reviews of Interventions [18] and presented based on Preferred Reporting Items for Systematic Reviews and Meta-analyses guidelines [19]. This protocol was registered with the International Platform of Registered Systematic Review and Meta-Analysis Protocols (INPLASY202050117).

### Literature search

Two researchers (W Ma and R Liu) searched the PubMed, Cochrane Library, EMBASE, China National Knowledge Infrastructure (CNKI), WANFANG DATA, Chinese Biomedical Literature (CBM), and Chongqing VIP (CQVIP) databases from their respective inception dates to April 19, 2020, using the keywords acupuncture, electroacupuncture and post-stroke depression to recognize published SRs and MAs evaluating the association between acupuncture and PSD. There were no language or study-blinding restrictions (detailed search strategies for PubMed are reported in Supplemental Table 1). We identified original randomized clinical trials (RCTs) included in the SRs/MAs. An additional search was conducted to identify recently published RCTs, from January 1, 2015, to May 12, 2020, meeting the inclusion criteria using the same databases and search strategy as described above (detailed search strategies for PubMed are reported in Supplemental Table 2).

### Inclusion and exclusion criteria

We abided by the Participants, Interventions, Comparisons, Outcomes and Study Design (PICOS) approach to establish the inclusion criteria, which were as follows: (1) Patients included were diagnosed with PSD with no restrictions on diagnostic criteria, gender, age, race, onset time or source of cases and regardless of the cause of the stroke. (2) Eligible interventions in the treatment group included acupuncture, electroacupuncture regardless of needling techniques, stimulation methods and acupoint selection. The control group was treated with sham acupuncture or conventional treatments (e.g., antidepressants, basic treatments, psychological counselling and physical rehabilitation). (3) The comparison reported was acupuncture/electroacupuncture vs. placebo/sham acupuncture, acupuncture/electroacupuncture vs. antidepressants, or acupuncture/electroacupuncture + conventional treatments vs. conventional treatments. (4) Degree of depression was selected as the targeted outcome because of its crucial role in PSD assessment [20]. The primary outcome included the HAM-D score, while the secondary outcome was adverse events. (5) The study was a RCT evaluating acupuncture for PSD.

The exclusion criteria were as follows: (1) duplicates, (2) literature with incorrect data or inaccessible data, and (3) Chinese trials that were not published in the Chinese core journal of Peking University (PKU).

### Study selection

There were two steps in the study selection process. First, we identified the eligible RCTs included in the SRs/MAs. Second, we recognized the RCTs of interest from the supplemental search. Both processes were performed by creating a database using Noteexpress 3.2.0 software, and after eliminating duplicates, we read the titles and abstracts for a preliminary screening. When studies could not be definitively excluded based on the titles and abstracts, we downloaded and the full text documents until all RCTs were confirmed. Two researchers (K Zhang and GW Cui) selected the literature, and disagreements were resolved by another reviewer (R Liu) when they had different opinions.

### Data extraction

Two researchers (GW Cui and QY Tong) independently extracted the following information using predesigned forms: lead author, publication year, details of intervention measurement, sample size and dropout, ages of the participants, degree of depression, depression duration, outcomes and adverse events. If the trials had more than two groups or permitted multiple comparisons, we extracted only the information and data of interest reported in the original articles. Any divergence was resolved by consensus (W Ma).

### Risk of bias assessment

The methodological quality of the included studies was assessed by two researchers (R Liu and K Zhang) according to the Cochrane handbook 5.1.0 [18], and the studies were ranked as high, unclear, and low risk in seven domains, namely randomization sequence generation, allocation concealment, blinding of participants and personnel, blinding of outcome assessment, incomplete outcome data, selective reporting and other bias. If necessary, we contacted the study authors when relevant information could not be identified from the articles or supplementary materials. In addition, we graded the overall quality of the included trials as high, moderate, or low quality according to previously published high-quality literature [21]. Disagreements were resolved through discussion with a third researcher (WD Shen).

### GRADE evaluation

The GRADE system [22] was used to evaluate the evidence quality of the evaluated outcomes. For each outcome, we automatically awarded each study four points, as these were RCTs, and they were downgraded if there were increased risks of bias, inconsistency, indirectness, inaccuracy, and publication bias. We classified evidence quality as A (high), B (moderate), C (low), or D (very low). Two researchers (R Liu and QY Tong) independently assessed the quality of evidence and resolved any disputes through discussions with a third researcher (WD Shen).

### Statistical analysis

We performed a meta-analysis of the RCTs with available data to calculate the effect size and 95% confidence interval (CI) using the random effects model based on significant heterogeneity (*P* < 0.1 and *I*^2^ > 50%). The HAM-D scale is presented as the mean difference (MD), as it is a continuous variable, and adverse events are presented as relative risks (RRs), as they are dichotomous variables. Statistical analyses were performed with RevMan 5.3 software. Two-sided *P* < 0.05 was considered statistically significant.

When possible and appropriate, planned subgroup analyses using the same models were conducted to identify the source of heterogeneity and reliability of the various results. Sensitivity analysis was performed by excluding low-quality studies and trials evaluating sham acupuncture. Publication bias was assessed by examining funnel plots for asymmetry when at least 10 articles were included.

## Results

### Literature selection

From the searches for SRs and MAs, 1037 potentially eligible records were identified. Titles and abstracts of these records were screened for exclusion. Full texts of 38 records were read, and 11 SRs/MAs that contained 167 RCTs met the inclusion criteria. After 141 RCTs were excluded for being a duplicate or being unpublished in the PKU journal, we obtained the full texts of 26 potential candidate RCTs. Among these, we ultimately included 13 articles. Supplemental Table 3 summarizes the RCTs included in the SRs/MAs.

**Fig. 1:**
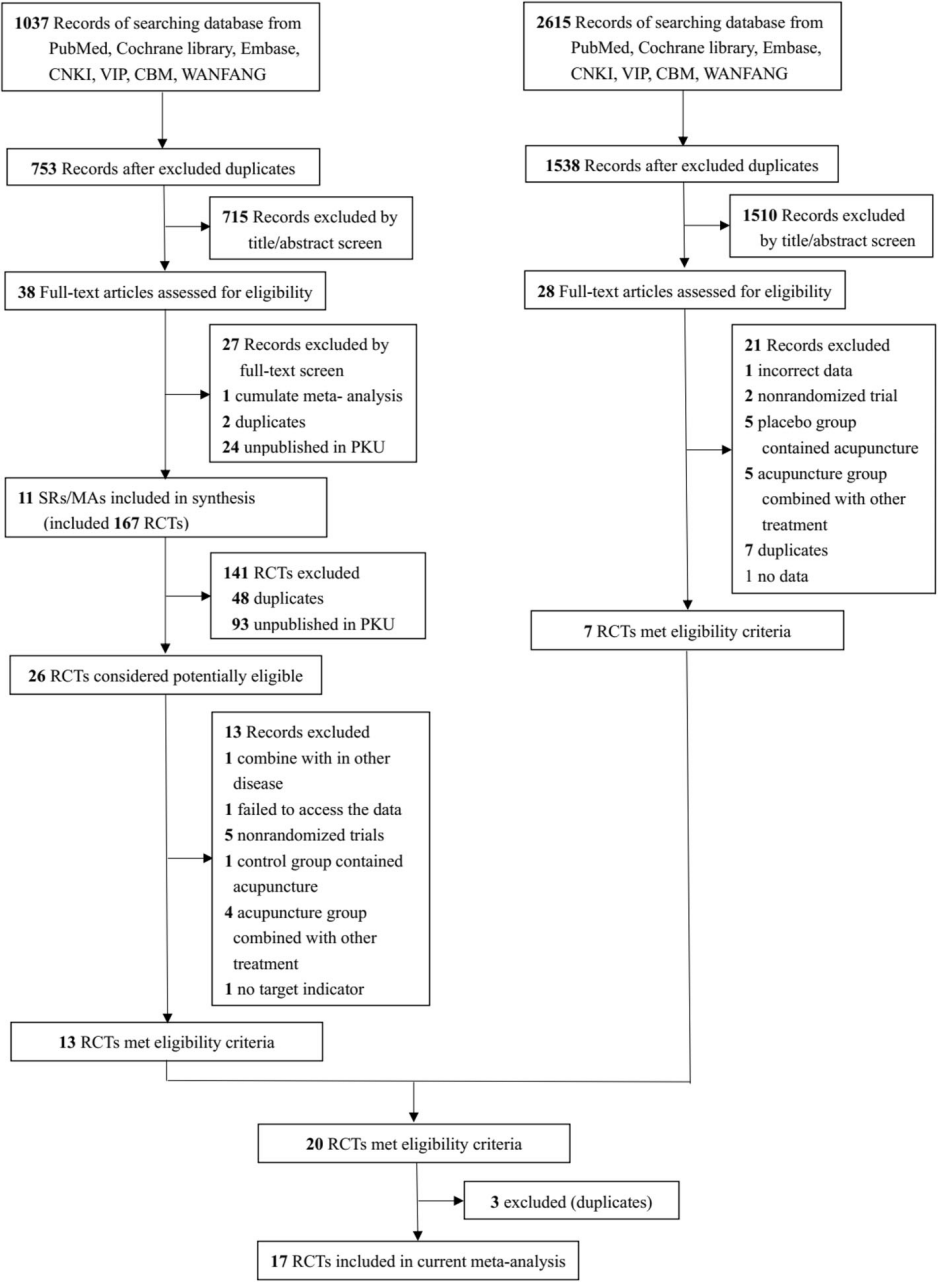
Flow diagram of the literature screening process and results

A total of 2615 articles were identified in a recent 5-year supplemental search; of these, 28 eligible full texts were reviewed after excluding duplicates and irrelevant articles. Seven trials met the inclusion criteria in this process. Finally, after excluding 3 duplicate trials, 17 RCTs involving 1402 participants were included in this review (Fig. 1). We excluded 33 trials for the reasons listed in Supplemental Table 4.

### Characteristics of the included studies

Quantitative synthesis was performed with 17 RCTs [16, 17, 23–37] by pooling the results in this review, and all trials were implemented in China. Sample sizes ranged from 10 to 145 participants, and a total of 1402 patients were included, with 683 (48.7%) in the experimental group and 719 (51.3%) in the control group. Seven trials [16, 17, 23, 26, 28, 35, 37] compared acupuncture or electroacupuncture combined with conventional treatment with conventional treatment, 11 studies [24, 25, 27, 29–34, 36, 37] compared acupuncture or electroacupuncture with antidepressants, and 2 [30, 33] adopted a 2-group parallel contrast design and compared the combination of real acupuncture and oral placebo with the combination of antidepressants and sham acupuncture. One study [37] was a 3-group design containing acupuncture alone, acupuncture combined with antidepressants and antidepressants alone. With regard to the degree of depression [38], 7 of the RCTs [23, 24, 26, 28, 31, 32, 35] recruited PSD participants with potential mild, moderate and severe depression, 10 studies [16, 17, 25, 27, 29, 30, 33, 34, 36, 37] enrolled patients with moderate and severe depression. The treatment durations ranged from 4 weeks to 6 months. The details of the duration, frequency, and number of sessions of the acupuncture programs are presented in the supplementary materials (Tables S6 ~ S8). For outcomes, we only evaluated the HAM-D scale because it is considered the “Gold Standard” in depression assessment [20]. Seven studies [25, 26, 28, 30, 33, 34, 37] used the HAM-D scale-17 items (HAM-D_17_) to evaluate the degree of depression, while four studies [16, 24, 27, 35] used the HAM-D scale-24 items (HAM-D_24_) as their outcome indicator. In addition, six trials [17, 23, 29, 31, 32, 36] did not report which version of the HAM-D they used. Adverse events are also an indispensable indicator to assess the safety of acupuncture. In this review, 10 studies [16, 17, 23, 29–31, 33, 34, 36, 37] reported adverse events; among them, three trials [16, 23, 36] found no obvious adverse events during the intervention, and one [33] was excluded because it presented overlapping data. Table 1 shows the details and characteristics of the included RCTs.

**Table 1:** Characteristics of RCTs Included in the Review and Summary of Trial Quality Assessment

Source	Reference No.	Intervention		Sample Size (T/C), Dropout (T/C)	Age (T/C)	Depression Definition	Duration	Outcome (s)	Adverse events	Overall quality for each trial
		Treatment	Control							
Acupuncture/EA + conventional treatment ^a^ vs. conventional treatment										
Guo 2009	[26]	Acupuncture (SP4, PC6, SJ5, GB41, SI3, BL62, LU7, KI6, 30 min/d) + psychological counselling+ physical rehabilitation	Sertraline (50 mg/d) + psychological counselling + physical rehabilitation	40/40	65.8 ± 9.61/67.6 ± 12.43	HAM-D_17_ ≥ 8	30 d	HAM-D_17_	NR	Moderate quality
Guo 2012	[28]	Acupuncture (PC6, GV 20, GV29, LR3, HT7, BL15, SP6, KI3, ST36, EX-HN1, ST40, 30 min/d) + physical rehabilitation	physical rehabilitation	55/55	60.30 ± 4.80/60.80 ± 3.90	HAM-D_17_ ≥ 8	30 d	HAM-D_17_	NR	Moderate quality
Zhang 2019	[17]	Acupuncture (LI4, ST40, ST16, HT7, SP6, LR3, PC6, EX-HN1, GV29, GV24, GV20, 40 min/d) + Fluoxetine (20 mg/d)	Fluoxetine (20 mg/d)	48/48	57.2 ± 8.5/58.5 ± 7.8	HAM-D ≥ 20	6 m	HAM-D scores	T: 2 cases of skin redness and swelling, 1 case of constipation. C: 1 case of constipation, 1 case of abdominal distension.	Moderate quality
Sun 2015	[35]	Acupuncture (GV 20, GV16, GV 24, GV 26, GV14, GV 11, 40 min/d) + Fluoxetine (20 mg/d)	Fluoxetine (20 mg/d)	33/30	59 ± 7/51 ± 6	HAM-D_24_ ≥ 8	4 w	HAM-D_24_	NR	Moderate quality
Chen 2018	[16]	Acupuncture (PC6, GV20, LI4, KI3, LR3, HT7, BL15, SP6, KI3, ST36, EX-HN1, ST10, 30 min/d) + conventional drug therapy	Conventional drug therapy	30/30	57 ± 11/58 ± 11	HAM-D_24_ ≥ 24	4 w	HAM-D_24_	None	Moderate quality
Jiang 2007	[23]	EA (GV20, EX-HN3, sparse-dense waves 2/100 Hz, tolerable strength, 30 min/d) + Fluoxetine (20 mg/d)	Fluoxetine (20 mg/d)	31/30, 3/2	60.32 ± 3.26/61. 18 ± 2.94	HAM-D scores ≥8	28 d	HAM-D scores	None	Moderate quality
Sun Y ^b^ 2015	[37]	EA (the midnight-noon ebb-flow theory, continuous wave, 2 Hz, 30 min/d) + Fluoxetine (20 mg/d)	Fluoxetine (20 mg/d)	31/31	67 ± 4/69 ± 5	HAM-D_17_ ≥ 17	6 m	HAM-D_17_	T: 1 case of dry mouth, 1 case of fatigue. C: 2 cases of intermittent headache, 2 cases of loss of appetite, 2 cases of nausea.	High quality
Acupuncture/EA (+placebo) vs. antidepressants (+sham acupuncture)										
Zhou 2012	[24]	Acupuncture (GV20, Ex-HN1, LR3, ST36, SP6, 20 min/d)	Fluoxetine (20 mg/d)	30/30, 2/2	65.34 ± 10.60/ 66.03 ± 9.51	HAM-D_24_ ≥ 8	30 d	HAM-D_24_	NR	Low quality
Sun 2013	[27]	Acupuncture (GV20, GV16, GV24, GV26, GV14, GV11, 40 min/d)	Fluoxetine (20 mg/d)	30/30	58 ± 8/59 ± 9	HAM-D_24_ ≥ 20	4 w	HAM-D_24_	NR	Moderate quality
Ding 2003	[29]	Acupuncture (GV20, DU24, DU16, 30 min/d)	Fluoxetine (20 mg/d)	30/30,1/1	NR	HAM-D score ≥ 20	60 d	HAM-D score	C: 3 cases of mild abdominal pain, 2 cases of mild nausea and vomiting, 3 cases of elevated GOT and GPT.	Moderate quality
Li 2011	[30]	Acupuncture (GV20, Ex-HN1, LR3, Ex-HN3, 30 min/d) + oral placebo	Non-acupoint spots+ Fluoxetine (20 mg/d)	23/20,1/2	29–90/32–63	HAM-D_17_ ≥ 17	6 w	HAM-D_17_	T: 1 case of pain, 1 case of dizziness and nausea and 1 case of haematoma. C: 1 case of dizziness, 1 case of numbness, 1 case of palpitation.	High quality
Liu 2006	[32]	Acupuncture (Ex-HN1, PC6, HT7, ST36, SP6, KI6, LR3, BL62, 30 min/d)	Fluoxetine (20 mg/d)	101/145	60.0 ± 9.8/59.6 ± 8.9	HAM-D scores ≥8	6 w	HAM-D score	NR	Moderate quality
Qian 2015	[33]	Acupuncture (GV26, PC6, HT7, ST36, 30 min/d) + placebo	Fluoxetine (20 mg/d) + Shallow puncture	32/33,2/1	67.59 ± 11.02/67.74 ± 8.61	HAM-D_17_ ≥ 17	6 w	HAM-D_17_	T: 2 cases of needle sticks, 1 case of bent needles, 2 cases of haematomas. C: 1 case of haematomas.	High quality
Zhang 2016	[34]	Acupuncture (GV26, GV24, GV15, BL18, BL23, LR3, KI3, PC6, HT7, CV17, 15 min/d)	Escitalopram Oxalate tablet (1.25–10 mg/d)	33/32,2/3	58.0 ± 5.3/59.0 ± 4.5	HAM-D_17_ ≥ 18	8 w	HAM-D_17_	C: 2 cases of dizziness, 2 cases of nausea and 1 case of tolerable joint pain	Moderate quality
Zhou 2016	[36]	Acupuncture (GV20, EX-HN1, HT7, SP6, PC6, GV24, 30 min/d)	Doxepin hydrochloride tablets	58/58	59.45 ± 9.03/58.16 ± 8.14	HAM-D scores ≥17	4 w	HAM-D scores	None	Low quality
Li 2015	[25]	EA (LI4, LR3, continuous waves 2 Hz, 20 min/d)	Fluoxetine (20 mg/d)	11/10	62.56 ± 6.85, 66.42 ± 6.25	HAM-D_17_ ≥ 18	8 w	HAM-D_17_	NR	Moderate quality
Chu 2007	[31]	EA (GV20, DU26, GV29, PC6, sparse-dense waves 6–8 Hz, 30 min/d)	Fluoxetine (20 mg/d)	36/36	54–78/58–72	HAM-D scores ≥8	8 w	HAM-D scores	C:3 cases of dry mouth, 3 cases of dizziness, 2 cases of drowsiness.	Moderate quality
Sun Y ^b^ 2015	[37]	EA (the midnight-noon ebb-flow theory, continuous wave, 2 Hz, 30 min/d)	Fluoxetine (20 mg/d)	31/31	67 ± 4/69 ± 5	HAM-D_17_ ≥ 17	6 m	HAM-D_17_	C: 2 cases of intermittent headache, 2 cases of loss of appetite, 2 cases of nausea.	High quality

*T* treatment group, *C* control group, *EA* electroacupuncture, *HAM-D* Hamilton Rating Scale for Depression, *HAM-D*_*17*_ HAM-D scale-17 items, *HAM-D*_*24*_ HAM-D scale-24 items, *NR* Not reporting

^a^Conventional treatment includes psychological counselling, physical rehabilitation, antidepressants and basic treatment; ^b^This trial contained 3 groups: acupuncture, acupuncture combined with antidepressants and antidepressants

### Risk of bias assessment

**Fig. 2:**
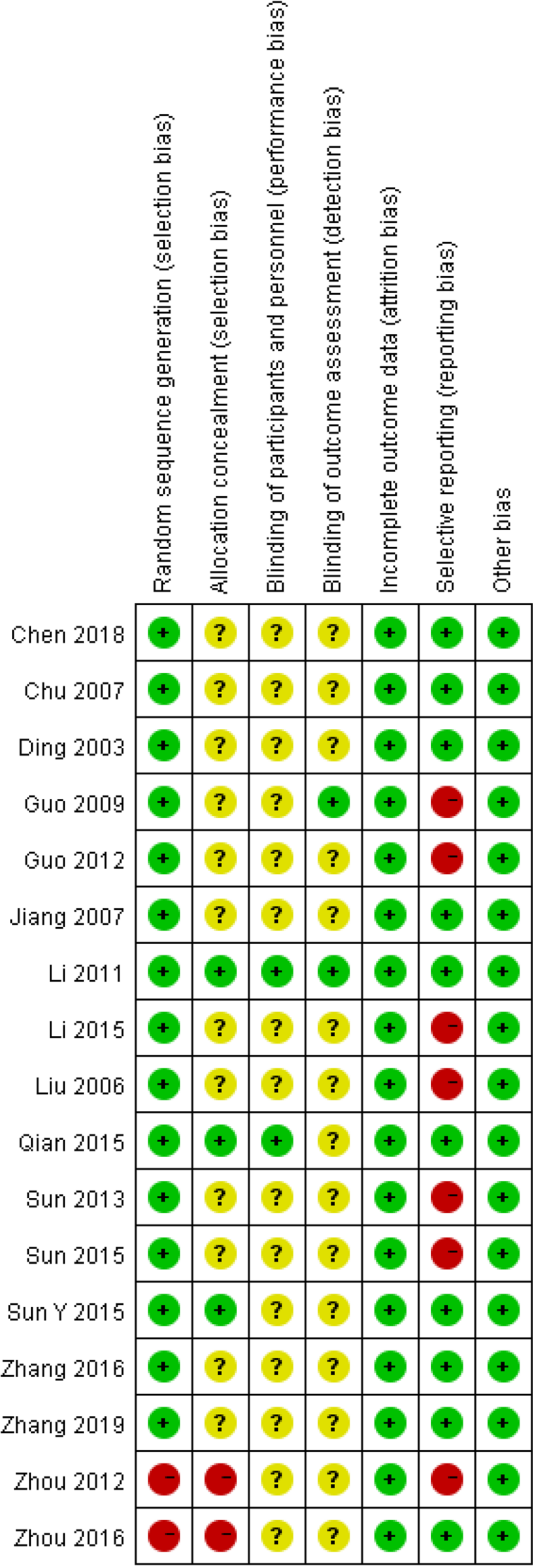
Risk of bias assessment for the 10 included studies

Fifteen studies [16, 17, 23, 25–35, 37] were rated as low risk because they reported specific randomization methods. However, two studies [24, 36] did not provide details regarding random sequence generation and allocation concealment, so they were both considered high risk. Three trials [30, 33, 37] that described adequate methods used for allocation concealment were ranked as low risk. Twelve studies [16, 17, 23, 25–29, 31, 32, 34, 35] had unclear risks in terms of allocation concealment. One study [30] reported a double-blinded trial design, and two additional studies [26, 33] reported that they blinded participants and outcome assessment reviewers; accordingly, they were ranked as low risk. All studies had dropout rates of less than 20%, so they were all rated as low risk in this aspect. Seven studies [24–28, 32, 35] failed to report adverse events; accordingly, they ranked as high risk in terms of reporting bias. Overall, three studies [30, 33, 37] were rated as high quality, two [24, 36] were rated as low quality, and the others were rated as moderate quality. Figure 2 shows the assessment of the risk of bias, and Table 1 shows the quality of the included trials.

### Outcomes

#### Ham-D_17_

**Fig. 3:**
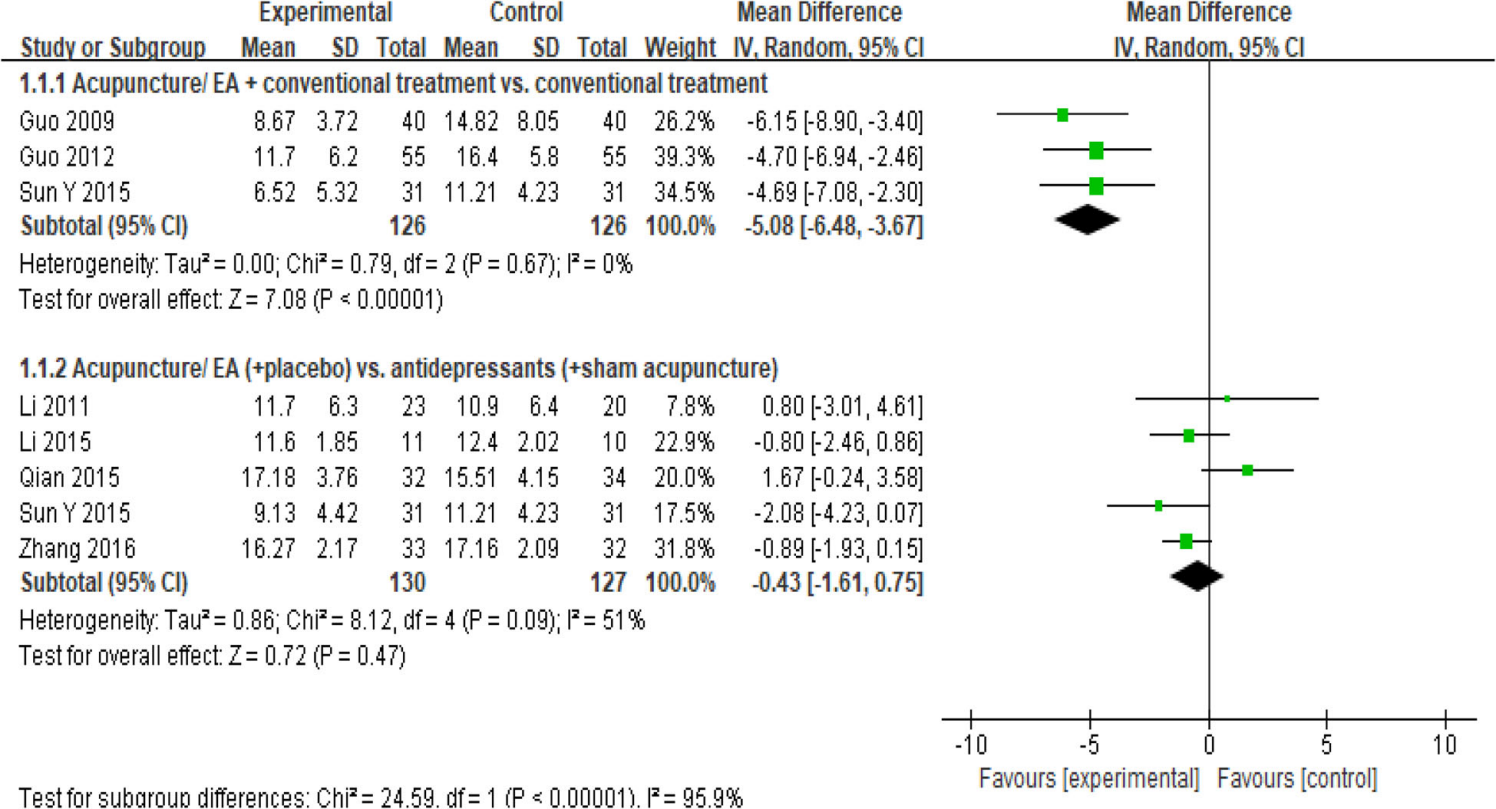
Forest plot for the HAM-D_17_ scale

A meta-analysis of 7 studies [25, 26, 28, 30, 33, 34, 37] included 509 participants who used the HAM-D_17_ to examine depression using a random-effects model. As shown in Fig. 3 studies [26, 28, 37] compared the combination of acupuncture/electroacupuncture and conventional treatment with conventional treatment and found that participants who received acupuncture exhibited significantly lower HAM-D_17_ scores than those in the control group (MD, − 5.08 [95% CI, − 6.48 to − 3.67], *I*^2^ = 0%). One trial [37] recruited participants with mild depression, and a sensitivity analysis in which this trial was excluded showed that the results did not change (Supplemental Table 5). Five studies [25, 30, 33, 34, 37] compared acupuncture/electroacupuncture (+placebo) with antidepressants (+sham acupuncture), and the subgroup results demonstrated that there was no significant difference (MD, − 0.43 [95% CI, − 1.61 to 0.75], *I*^2^ = 51%). Because Qian 2015 [33] and Li 2011 [30] combined treatment with placebo, we excluded them in the sensitivity analysis, and the result was overturned (Supplemental Table 5).

#### Ham-D_24_

**Fig. 4:**
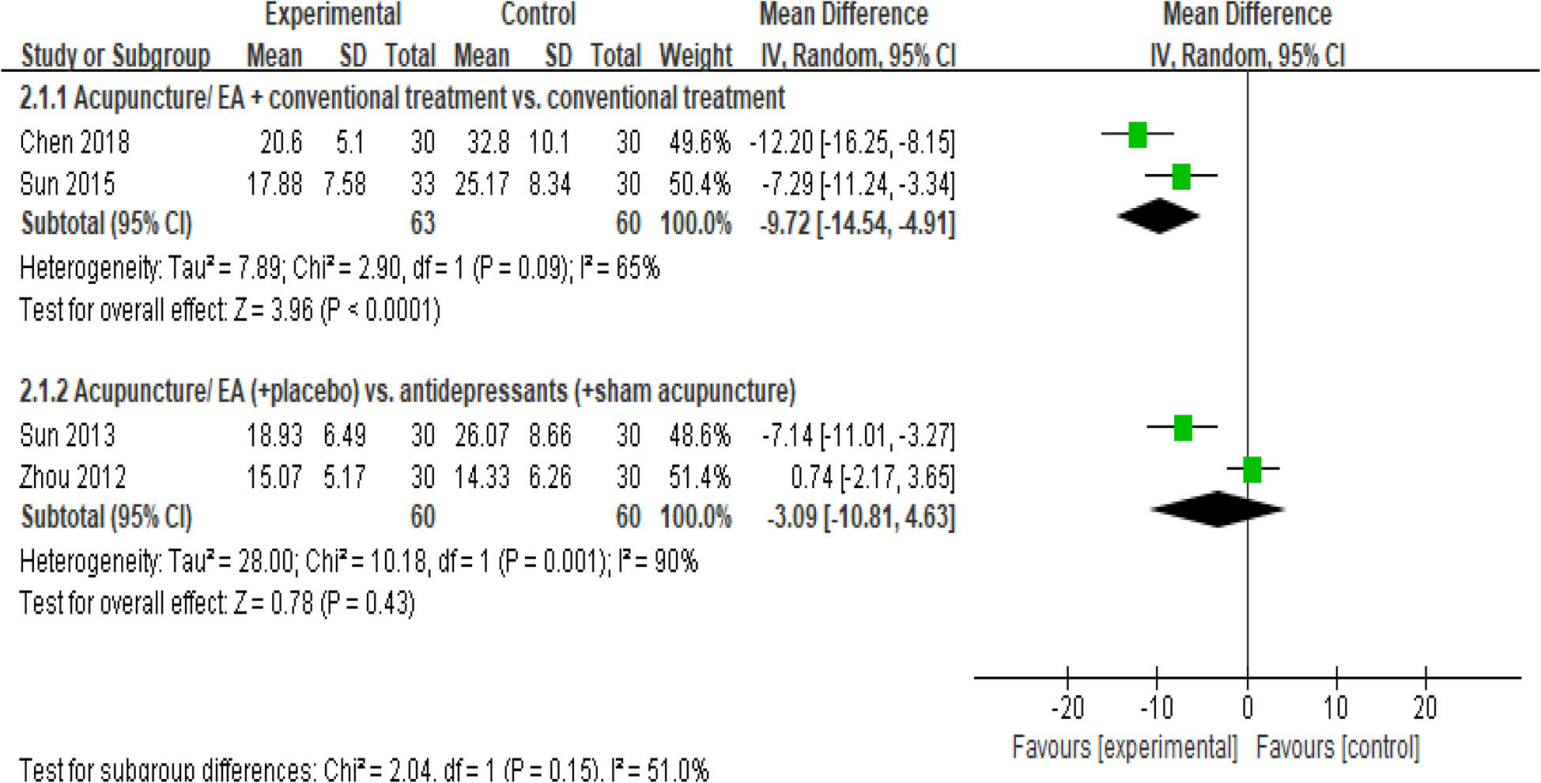
Forest plot for the HAM-D_24_ scale

We identified 4 studies [16, 24, 27, 35] that made use of HAM-D_24_ tool to measure depression (Fig. 4). Two studies [16, 35] compared the combination of acupuncture/electroacupuncture and conventional treatment with conventional treatment, and a pooled analysis demonstrated that acupuncture led to significant reductions in HAM-D_24_ scores relative to sham acupuncture (MD, − 9.72 [95% CI, − 14.54 to − 4.91], *I*^2^ = 65%). Another two studies compared acupuncture/electroacupuncture with antidepressants, and there was no significant difference in the risk of depression between these treatments (MD, − 3.09 [95% CI, − 10.81 to 4.63], *I*^2^ = 90%).

#### Ham-d

**Fig. 5:**
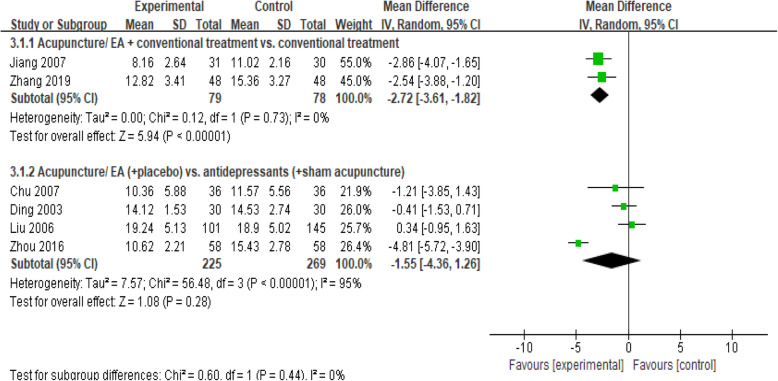
Forest plot for the HAM-D scale

Six studies [17, 23, 29, 31, 32, 36] did not report which version of the HAM-D they used (Fig. 5). Pooled results from 2 studies [17, 23] showed that the reduction in HAM-D score was significantly associated with a combination of acupuncture and conventional treatment when compared with conventional treatment (MD, − 2.72 [95% CI, − 3.61 to − 1.82], I2 = 0%). Four studies [29, 31, 32, 36] showed a greater association between acupuncture and HAM-D than between antidepressants and HAM-D, though the studies had substantial heterogeneity (MD, − 1.55 [95% CI, − 4.36 to 1.26], *I*^2^ = 95%). In the sensitivity analyses, excluding the low-quality study [36] and combining studies by degree of depression did not change the results (Supplemental Table 5).

#### Adverse events

**Fig. 6:**
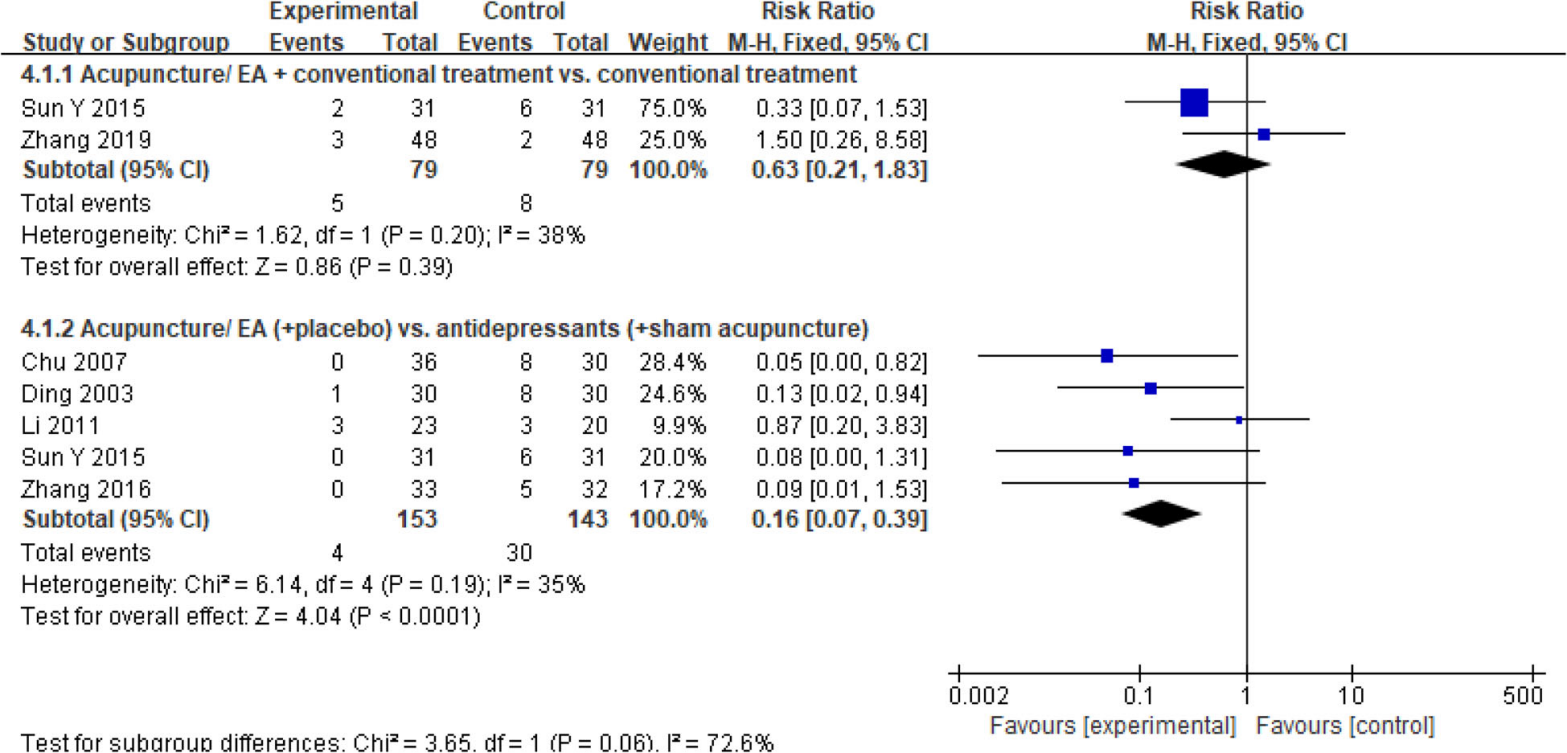
Forest plot of the adverse events

Ten studies [16, 17, 23, 29–31, 33, 34, 36, 37] reported adverse events during the study period (Fig. 6). Data from 4 RCTs [29–31, 34] were abstract to show that the occurrence of adverse events was significantly associated with acupuncture when compared with antidepressants (RR, 0.16 [95% CI, 0.07 to 0.39], *I*^2^ = 35%). When Li 2011 was excluded [30], sensitivity analyses showed that the results were not altered (Supplemental Table 5). There was no significant difference between acupuncture combined with conventional treatment and conventional treatment (RR, 0.63 [95% CI, 0.21 to 1.83], *I*^2^ = 38%). In addition, the adverse events reported were mild and disappeared without medical or specific intervention.

#### Quality of evidence

According to the GRADE system, the 8 outcomes were ranked as having low- or very low-quality evidence. The factors driving this quality assessment might be inappropriate randomization methods, lack of allocation concealment, and small sample size. The details of the evidence quality assessment are shown in Table 2.

**Table 2:** Quality of evidence of included reviews according to GRADE

Certainty Assessment							No. of Patients			
No. of Studies	Reference No.	Risk of Bias	Inconsistency	Indirectness	Imprecision	Publication Bias	Treatment Group	Control Group	Effect (95% CL)	Certainty
Acupuncture/EA (+placebo) compared to antidepressants (+sham acupuncture)										
HAM-D_17_										
5	[25, 30, 33, 34, 37]	serious ^a^	serious ^b^	not serious	serious ^c^	none	130	127	MD −0.43 (−1.61, 0.75)	⨁◯◯◯VERY LOW
HAM-D_24_										
2	[24, 27]	serious ^a^	serious ^d^	not serious	very serious ^c^	none	60	60	MD −3.09 (− 10.81, 4.63)	⨁◯◯◯VERY LOW
HAM-D										
4	[29, 31, 32, 36]	serious ^a^	serious ^e^	not serious	not serious	none	225	269	MD − 1.55 (−4.36, 1.26)	⨁⨁◯◯LOW
Adverse events										
5	[29–31, 34, 37]	serious ^a^	not serious	serious ^f^	serious ^c^	none	4/153 (2.6%)	30/143 (21%)	RR 0.16 (0.07, 0.39)	⨁◯◯◯VERY LOW
Acupuncture/EA + conventional treatment compared to conventional treatment										
HAM-D_17_										
3	[26, 28, 37]	serious ^a^	not serious	not serious	serious ^c^	none	126	126	MD −5.08 (−6.4, − 3.67)	⨁⨁◯◯LOW
HAM-D_24_										
2	[16, 35]	serious ^a^	serious ^g^	not serious	very serious ^c^	none	63	60	MD −9.72 (−14.54, − 4.91)	⨁◯◯◯VERY LOW
HAM-D										
2	[17, 24]	serious ^a^	not serious	not serious	very serious ^c^	none	79	78	MD −**2.72** (−3.61, − 1.82)	⨁◯◯◯VERY LOW
Adverse events										
2	[17, 37]	serious ^a^	not serious	serious ^f^	very serious ^c^	none	5/79 (6.3%)	8/79 (10.1%)	RR 0.66 (0.15,2.89)	⨁◯◯◯VERY LOW

*CI* confidence interval, *MD* mean difference, *RR* relative risk, *HAM-D* Hamilton Depression Scale, *HAM-D*_*17*_ HAM-D scale-17 items, *HAM-D*_*24*_ HAM-D scale-24 items

^a^This trial had a large bias in randomization, allocation concealment, blinding, or selective reporting; ^b^I^2^ = 51%; ^c^Small sample size; ^d^I^2^ = 90%; ^e^I^2^ = 95%; ^f^This is a secondary outcome; ^g^I^2^ = 65%

## Discussion

### Main findings

This MA of 17 RCTs involving 1402 patients with PSD illustrated that acupuncture combined with conventional treatment could significantly reduce the PSD degree, regardless of which versions of the HAM-D score is used, compared with conventional treatment, although the studies have a low to very low level of evidence. However, of the comparison of acupuncture and antidepressants was inconclusive, and the quality of evidence was low to very low. We refined the different groups to determine the incidence of adverse events. Acupuncture was safer than antidepressants, and the combination of acupuncture and conventional treatment had no significant difference in adverse events compared with conventional treatment.

### Comparison to previous studies

This nonblinded review of trials highlights that the combination of acupuncture and conventional treatment had a significant effect on HAM-D scores compared with conventional treatment, and the result was consistent regardless of the degree of depression, which is consistent with findings in previous studies and reviews [39, 40] and helps to increase confidence in our findings. Although evidence from open-label studies illustrates the increased risk of bias from nonblinding, the use of nonblinded pragmatic trial procedures to maximize the applicability of treatment effectiveness in real-world situations has increased in recent years [41, 42]. Apart from this, we found that acupuncture has no effect on reducing the degree of PSD compared with antidepressants; these results are contrary to those reported in a previous study [43]. When exploring the reasons for this discrepancy, we found that the placebo effect [44] is an important factor that cannot be ignored. Placebo interventions aim to intentionally utilize the placebo effect by increasing patients’ expectations [45]. A meta-analysis demonstrated that sham acupuncture (SA) had a larger effect than other placebos [46]. Thus, it was very difficult to evaluate the effect of SA compared to that of real acupuncture [47]. Therefore, when we removed the placebo-controlled study, we found that the efficacy of acupuncture for PSD might be better than that of antidepressants.

In this review, we also focused on the differences in the estimated efficacy of acupuncture on PSD using different versions of the HAM-D scale. Our review included the HAM-D_17_, HAM-D_24_ and other versions, which are all commonly used clinically [12]. The HAM-D_17_ is sensitive enough to discriminate different levels of depression severity and a cut-off score of < 8 defines no depression [48, 49]. For the HAM-D_24_, a cut-off score of less than 10 is used; this version has expanded items assessing worthlessness, symptoms of helplessness, hopelessness and cognitive impairment [48], though it was less sensitive with regard to discriminating antidepressants and placebo than the HAM-D_17_ [50]. A study [51] has indicated that when compared with antidepressants, acupuncture had lower scores on the HAM-D_17_ than on the HAM-D_24_. In our review, the comparison of acupuncture and antidepressants showed no difference in efficacy when different versions of the HAM-D were used, so we could not make a more appropriate version to evaluate the efficacy of acupuncture on PSD. In our study, acupuncture combined with conventional treatments compared with conventional treatments for PSD was the most effective when depression was measured using the HAM-D_24_ scale, followed by the HAM-D_17_ scale, finally, the HAM-D scale. There exists a weak relationship between the HAM-D scale version used to evaluate the effect of acupuncture on depression and the strength of the association, yet this relationship depended on the intervention used. In addition, some researchers have proposed that the Post-Stroke Depression Scale (PSDS) is a valid, reliable and specific tool to evaluate PSD [52], and some hold that the HAM-D_6_ could be a new indicator to evaluate the effect of acupuncture on PSD because it aims to investigate the discrimination between active treatment and placebo [50, 53]. Therefore, deciding which version of the HAM-D scale to use remains a challenge, but in terms of redundancy, the HAM-D_24_ clearly carries more load than the HAM-D_17_ [50], and in terms of usage, the HAM-D_17_ (41%) was more common in our review than HAM-D_24_ (23%).

A recent series of studies of antidepressants have claimed that antidepressant effectiveness is beyond dispute, but its side effects should not be ignored [54–56]. In our review, we prove that the combination of acupuncture and antidepressants has no significant effect on adverse events compared with antidepressants alone. However, the incidence of adverse events associated with acupuncture was apparently lower than that associated with antidepressants, and the results of sensitivity analyses were not altered when studies with placebo were excluded. This suggests that acupuncture may be a safer treatment for PSD. This finding is similar to that of a previous review [11].

One common issue in acupuncture programs is the lack of standardization of point selection, duration, frequency, number of sessions and use of electroacupuncture [57]. The 17 RCTs evaluated here revealed that the most commonly selected points were GV20 (65%), PC6 (53%), and LR3 (47%), which is different from the most recent study [11]. Additionally, our review varied greatly in terms of acupuncture duration and the number of sessions, ranging from 15 min to 30 min of duration and from 1 month to 6 months in terms of number of sessions. There is no clear concept of an appropriate acupuncture dose and how much treatment is needed for a given situation. The lack of agreement on the optimal acupuncture treatment for any particular condition may partly impact the acupuncture effect [58].

### Strengths and limitations

There are some advantages of this review. To ensure the quality of the included studies, we used more rigorous inclusion criteria; for example, all Chinese RCTs included in this review were published in the PKU journal. In addition, considerable effort was made to carry out an extensive literature search, including relevant SRs and MAs, as well as a supplemental search of recent RCTs. For intervention methods, we made efforts to analyse the combination of acupuncture and conventional treatment compared with conventional treatment or acupuncture compared with antidepressants. In addition, we investigated the effects of acupuncture for PSD using different HAM-D versions, which could provide a clinical reference for further study.

This review has several limitations. First, due to the quantities of included trials, we could not explore whether different acupuncture durations could impact the results. Second, this review indicated that acupuncture for PSD can largely reduce the degree of depression, but we did not find a suitable clinically important difference (MCID) for the HAM-D, which limits our ability to determine the clinical effectiveness. Third, some of the studies included in this review may reduce the reliability of the conclusions due to factors such as a lack of allocation concealment, small sample size, or unclear study blinding.

## Conclusions

This review strengthens the evidence that acupuncture could reduce the degree of PSD. However, there is still a lack of moderate and high quality evidence to support this conclusion. Further methodologically rigorous and adequately powered primary studies are necessary to assess the effect of acupuncture on PSD.

## Supplementary Information

**Additional file 1:** **Table S1.** Search strategy to identify RCTs in PubMed. **Table S2.** Search strategy to identify meta-analysis in PubMed. **Table S3.** Randomized trials included in systematic reviews or meta-analyses evaluating acupuncture and PSD. **Table S4.** Excluded trials and reasons for exclusion. **Table S5.** Results of sensitivity analyses excluding the listed trials. **Table S6.** The acupoints and their frequency of use in the included studies. **Table S7.** The treatment time and frequency in the included studies. **Table S8.** The treatment time and frequency in the included studies.

## Data Availability

The datasets generated and analysed during the current study are available in the 7 databases (details in the study selection section).

## Abbreviations

PSD: Post-stroke depression
RCTs: Randomized clinical trials
SRs: Systematic reviews
MAs: Meta-analyses
GRADE: Grades of Recommendation, Assessment, Development and Evaluation
5-HT: 5-hydroxytryptamine
HAM-D: Hamilton Rating Scale for Depression
CNKI: China National Knowledge Infrastructure
CBM: Chinese Biomedical Medicine disc
CQVIP: Chongqing VIP
CI: Confidence interval
MD: Mean difference
RR: Relative risk
PSDS: Post-Stroke Depression Scale
T: Treatment group
C: Control group
EA: Electroacupuncture
