# Supplementary material for: Acupuncture for post-stroke depression: a systematic review and meta-analysis
Source: BMC Complement Med Ther. 2021 Apr 1;21:109. doi: 10.1186/s12906-021-03277-3 (PMC8017746; doi:10.1186/s12906-021-03277-3)
Supplement: Supplementary file 1 — Additional file 1: Table S1. Search strategy to identify RCTs in PubMed. Table S2. Search strategy to identify meta-analysis in PubMed. Table S3. Randomized trials included in systematic reviews or meta-analyses evaluating acupuncture and PSD. Table S4. Excluded trials and reasons for exclusion. Table S5. Results of sensitivity analyses excluding the listed trials. Table S6. The acupoints and their frequency of use in the included studies. Table S7. The treatment time and frequency in the included studies. Table S8. The treatment time and frequency in the included studies. [file 12906_2021_3277_MOESM1_ESM.docx]

**Table S1** Search strategy to identify RCTs in PubMed

| **Database** | **Search Strategy** | **Items** |
| --- | --- | --- |
| **PubMed** | #1 “Depression” [Mesh] | 117190 |
|  | #2 “Stroke” [Mesh] | 132698 |
|  | #3 #1AND #2 | 1692 |
|  | #4 ((((((depression [Title/Abstract]) OR stroke [Title/Abstract]) OR post stroke [Title/Abstract]) OR after stroke [Title/Abstract]) OR post-stroke depression [Title/Abstract]) OR post stroke depression [Title/Abstract]) OR PSD | 572423 |
|  | #5 #3 OR #4 | 572495 |
|  | #6 “Acupuncture” [Mesh] OR “Acupuncture Therapy” [Mesh] | 24890 |
|  | #7 ((Acupuncture [Title/Abstract]) OR Electroacupuncture [Title/Abstract]) OR scalp needle [Title/Abstract] | 24570 |
|  | #8 #6 OR #7 | 30684 |
|  | #9 #5 AND #8 | 1931 |
|  | #10 #9 AND Publication date from 2015/01/01 to 2020/05/12 | 867 |
|  | #11 #10 AND Clinical trial | 240 |

**Table S2** Search strategy to identify meta-analysis in PubMed

| **NO.** | **Search strategy** | **Items** |
| --- | --- | --- |
| #1 | Search "Depression"[Mesh] | 116604 |
| #2 | Search "Stroke"[Mesh] | 132098 |
| #3 | #1 AND #2 | 1679 |
| #4 | Search ((((((depression[Title/Abstract]) OR stroke[Title/Abstract]) OR post stroke[Title/Abstract]) OR after stroke[Title/Abstract]) OR post-stroke depression[Title/Abstract]) OR post stroke depression[Title/Abstract]) OR PSD[Title/Abstract] | 569867 |
| #5 | #3 OR #4 | 569938 |
| #6 | Search "Acupuncture"[Mesh] OR "Acupuncture Therapy"[Mesh] | 24831 |
| #7 | Search ((Acupuncture*[Title/Abstract]) OR Electroacupuncture[Title/Abstract]) OR scalp needle[Title/Abstract] | 24456 |
| #8 | #6 OR #7 | 30562 |
| #9 | Search "Meta-Analysis" [Publication Type] OR "Meta-Analysis as Topic"[Mesh] | 130766 |
| #10 | Search ((Meta-Analysis[Title/Abstract]) OR Meta analys*[Title/Abstract]) OR Systematic review*[Title/Abstract] | 261411 |
| #11 | #9 OR #10 | 286561 |
| #12 | #5 AND #8 AND #11 | 219 |

| **Table S3** Randomized trials included in systematic reviews or meta-analyses evaluating acupuncture and PSD | | | | | | | | | | | |
| --- | --- | --- | --- | --- | --- | --- | --- | --- | --- | --- | --- |
| **Trials** | **Systematic reviews or meta-analyses** | | | | | | | | | | |
|  | Li 2017 ^1^ | Zhang 2014 ^2^ | Zhang 2012 ^3^ | Zhang 2009 ^4^ | Zhang 2010 ^5^ | Zhang 2019 ^6^ | Zhan 2016 ^7^ | Li 2012 ^8^ | Zhou 2018 ^9^ | Xu 2019 ^10^ | Zhang W 2014 ^11^ |
| Bi 2010 ^12^ | Y |  |  |  |  |  |  | Y |  |  |  |
| Chen 2012 ^13^ | Y |  |  |  |  |  |  |  |  |  |  |
| Chen 2005 ^14^ | Y |  | Y |  |  |  | Y | Y |  |  |  |
| Chu 2007 ^15^ | Y |  |  |  | Y |  |  | Y |  |  | Y |
| Dong 2007 ^16^ | Y |  | Y | Y |  |  | Y | Y | Y |  | Y |
| Guo 2011 ^17^ | Y |  |  |  |  |  | Y |  |  |  |  |
| Hong 2015 ^18^ | Y |  |  |  |  |  |  |  |  |  |  |
| Huang 2014 ^19^ | Y |  |  |  |  |  | Y |  |  |  |  |
| Kang 2014 ^20^ | Y |  |  |  |  |  |  |  |  |  |  |
| Li 2015 ^21^ | Y |  |  |  |  |  |  |  |  |  |  |
| Li 2013 ^22^ | Y |  |  |  |  |  | Y |  |  |  |  |
| Long 2004 ^23^ | Y |  |  |  |  |  |  |  |  |  |  |
| Peng 2011 ^24^ | Y |  |  |  |  |  | Y |  |  |  |  |
| Wang 2015 ^25^ | Y |  |  |  |  |  |  |  |  |  |  |
| Xu 2015 ^26^ | Y |  |  |  |  |  |  |  |  |  |  |
| Zhou 2007 ^27^ | Y |  |  |  |  |  | Y | Y |  |  |  |
| Zhou 2010 ^28^ | Y |  |  |  |  |  | Y | Y | Y |  |  |
| Zhu 2012 ^29^ | Y |  |  |  |  |  |  |  | Y |  |  |
| Guo 2009 ^30^ |  | Y |  |  |  |  |  |  |  |  | Y |
| Chi 2011 ^31^ |  | Y |  |  |  |  |  |  |  |  | Y |
| He 2006 ^32^ |  | Y |  |  | Y |  |  |  |  |  |  |
| Zheng 2010 ^33^ |  | Y |  |  |  |  |  |  |  |  |  |
| Yuan 2006 ^34^ |  | Y |  |  |  |  |  |  |  |  |  |
| Yang 2003 ^35^ |  | Y |  |  |  |  |  |  |  |  |  |
| Ding 2003 ^36^ |  | Y | Y |  | Y |  |  |  |  |  |  |
| Zhou 2012 ^37^ |  | Y |  |  |  |  |  |  |  |  | Y |
| Li 2011 ^38^ |  | Y |  |  |  | Y |  |  | Y |  |  |
| Li 2009 ^39^ |  | Y |  |  |  |  |  |  |  |  |  |
| Liu 2008 ^40^ |  | Y |  |  |  |  |  |  |  |  |  |
| Ma 2012 ^41^ |  | Y |  |  |  |  |  |  |  |  |  |
| Sun 2010 ^42^ |  | Y |  |  |  |  |  |  |  |  |  |
| Sun 2012 ^43^ |  | Y |  |  |  |  |  |  |  |  |  |
| Wang 2010 ^44^ |  | Y |  |  |  |  |  |  |  |  |  |
| Zhu2008 ^45^ |  | Y |  |  |  |  |  |  |  |  |  |
| He 2009 ^46^ |  | Y |  |  |  |  |  |  |  |  | Y |
| Zhang 2004 ^47^ |  |  | Y | Y |  |  |  |  |  |  |  |
| Sun 2004 ^48^ |  |  | Y |  |  |  |  |  |  |  |  |
| Dai 2009 ^49^ |  |  | Y |  |  |  |  |  |  |  |  |
| He 2007 ^50^ |  |  | Y |  |  |  |  |  |  |  |  |
| Jiang 2006 ^51^ |  |  | Y |  |  |  |  |  |  |  |  |
| Li Y 2009 ^52^ |  |  | Y |  |  |  |  |  |  |  |  |
| Wang 2004 ^53^ |  |  | Y | Y |  |  |  |  |  |  |  |
| Zhang 2009 ^54^ |  |  | Y |  |  |  |  |  |  |  |  |
| Wei 2008 ^55^ |  |  | Y |  |  |  |  |  |  |  |  |
| Wang 2003 ^56^ |  |  | Y | Y | Y |  |  |  |  |  |  |
| Zhuang 2004 ^57^ |  |  | Y | Y |  |  |  |  |  |  |  |
| Zhang Y 2004 ^58^ |  |  | Y |  |  |  |  |  |  |  |  |
| Jiang 2007 ^59^ |  |  |  | Y |  |  |  |  |  |  |  |
| Cui 2005 ^60^ |  |  |  | Y |  |  |  |  |  |  |  |
| Nie 2006 ^61^ |  |  |  | Y |  |  |  | Y |  |  | Y |
| Xing 2005 ^62^ |  |  |  | Y |  |  |  |  |  |  |  |
| Wang Y 2004 ^63^ |  |  |  |  | Y |  |  |  |  |  |  |
| Li 2004 ^64^ |  |  |  | Y |  |  |  |  |  |  |  |
| Liu 2006 ^65^ |  |  |  | Y |  |  |  |  |  |  |  |
| Kong 2007 ^66^ |  |  |  | Y |  |  |  |  |  |  |  |
| Wang 2006 ^67^ |  |  |  | Y |  |  |  |  |  |  |  |
| Gu 2005 ^68^ |  |  |  | Y | Y |  |  |  |  |  |  |
| Yang 2007 ^69^ |  |  |  | Y | Y |  |  |  |  |  |  |
| Li 2007 ^70^ |  |  |  | Y |  |  |  |  |  |  |  |
| Zhang 2007 ^71^ |  |  |  | Y |  |  |  |  |  |  |  |
| Cheng 2007 ^72^ |  |  |  |  | Y |  |  |  |  |  |  |
| Huang 2004 ^73^ |  |  |  |  | Y |  |  |  |  |  |  |
| Peng 2007 ^74^ |  |  |  |  | Y |  |  |  |  |  |  |
| Tang 2003 ^75^ |  |  |  |  | Y |  |  |  |  |  |  |
| Yin 2004 ^76^ |  |  |  |  | Y |  |  |  |  |  |  |
| Zhai 2004 ^77^ |  |  |  |  | Y |  |  |  |  |  |  |
| Zhao 2007 ^78^ |  |  |  |  | Y |  |  |  |  |  |  |
| Liu 2013 ^79^ |  |  |  |  |  | Y |  |  | Y |  |  |
| Cao 2014 ^80^ |  |  |  |  |  | Y |  |  |  | Y |  |
| Liao 2017 ^81^ |  |  |  |  |  | Y |  |  |  |  |  |
| Chu 2017 ^82^ |  |  |  |  |  | Y |  |  |  |  |  |
| Yan 2018 ^83^ |  |  |  |  |  | Y |  |  |  |  |  |
| Sun 2015 ^84^ |  |  |  |  |  | Y | Y |  | Y |  |  |
| Zhang 2013 ^85^ |  |  |  |  |  |  | Y |  |  |  |  |
| Huang 2005 ^86^ |  |  |  |  |  |  | Y | Y |  |  |  |
| Yao 2010 ^87^ |  |  |  |  |  |  | Y |  |  |  |  |
| Wang Y 2010 ^88^ |  |  |  |  |  |  | Y | Y |  |  |  |
| Chen 2009 ^89^ |  |  |  |  |  |  | Y |  |  |  |  |
| You 2004 ^90^ |  |  |  |  |  |  |  | Y |  |  |  |
| Xin 2008 ^91^ |  |  |  |  |  |  |  | Y |  |  | Y |
| Chen 2011 ^92^ |  |  |  |  |  |  |  | Y |  |  |  |
| Wang 2011 ^93^ |  |  |  |  |  |  |  | Y |  |  |  |
| Wu 2011 ^94^ |  |  |  |  |  |  |  |  | Y |  |  |
| Liu 2012 ^95^ |  |  |  |  |  |  |  |  | Y |  |  |
| Shan 2017 ^96^ |  |  |  |  |  |  |  |  | Y |  |  |
| Kong 2015 ^97^ |  |  |  |  |  |  |  |  | Y |  |  |
| Zhang 2015 ^98^ |  |  |  |  |  |  |  |  | Y |  |  |
| Zhu 2014 ^99^ |  |  |  |  |  |  |  |  | Y |  |  |
| Yang 2013 ^100^ |  |  |  |  |  |  |  |  | Y |  |  |
| Duan 2014 ^101^ |  |  |  |  |  |  |  |  | Y |  |  |
| Wang G 2015 ^102^ |  |  |  |  |  |  |  |  | Y |  |  |
| Wang 2016 ^103^ |  |  |  |  |  |  |  |  | Y |  |  |
| Nie 2013 ^104^ |  |  |  |  |  |  |  |  | Y |  |  |
| Lu 2013 ^105^ |  |  |  |  |  |  |  |  | Y |  |  |
| Chen 2016 ^106^ |  |  |  |  |  |  |  |  | Y |  |  |
| Chen 2018 ^107^ |  |  |  |  |  |  |  |  |  | Y |  |
| Feng 2018 ^108^ |  |  |  |  |  |  |  |  |  | Y |  |
| Guo 2012 ^109^ |  |  |  |  |  |  |  |  |  | Y |  |
| Jiao 2017 ^110^ |  |  |  |  |  |  |  |  |  | Y |  |
| Jing 2018 ^111^ |  |  |  |  |  |  |  |  |  | Y |  |
| Li 2018 ^112^ |  |  |  |  |  |  |  |  |  | Y |  |
| Liu 2014 ^113^ |  |  |  |  |  |  |  |  |  | Y |  |
| Sun P 2015 ^114^ |  |  |  |  |  |  |  |  |  | Y |  |
| Sun X 2018 ^115^ |  |  |  |  |  |  |  |  |  | Y |  |
| Sun Y 2015 ^116^ |  |  |  |  |  |  |  |  |  | Y |  |
| Ye 2013 ^117^ |  |  |  |  |  |  |  |  |  | Y |  |
| Tong 2012 ^118^ |  |  |  |  |  |  |  |  |  | Y |  |
| Wang 2018 ^119^ |  |  |  |  |  |  |  |  |  | Y |  |
| Wu 2008 ^120^ |  |  |  |  |  |  |  |  |  | Y |  |
| Xia 2015 ^121^ |  |  |  |  |  |  |  |  |  | Y |  |
| Yao 2018 ^122^ |  |  |  |  |  |  |  |  |  | Y |  |
| Yu 2010 ^123^ |  |  |  |  |  |  |  |  |  | Y |  |
| Gao 2013 ^124^ |  |  |  |  |  |  |  |  |  | Y |  |
| Nie 2012 ^125^ |  |  |  |  |  |  |  |  |  |  | Y |
| Wang L 2011 ^126^ |  |  |  |  |  |  |  |  |  |  | Y |
| Fu 2013 ^127^ |  |  |  |  |  |  |  |  |  |  | Y |
| Nie 2011 ^128^ |  |  |  |  |  |  |  |  |  |  | Y |
| Sun 2013 ^129^ |  |  |  |  |  |  |  |  |  |  | Y |
| He J 2007 ^130^ |  |  |  |  | Y |  |  |  |  |  |  |

Note: Y, yes (Each “Y” indicates that this trial was included in the systematic reviews or meta-analyses of corresponding column)

**References**

1. Li XB, Wang J, Xu AD*, et al.* Clinical effects and safety of electroacupuncture for the treatment of post-stroke depression: a systematic review and meta-analysis of randomized controlled trials. *Acupuncture in medicine*, 2018,36(5):284-293.
2. Zhang JP, Chen J, Chen JQ*, et al.* Early filiform needle acupuncture for poststroke depression: a meta-analysis of 17 randomized controlled clinical trials. *Neural Regeneration Research*, 2014,9(7): 773-784.
3. Zhang GC, Fu WB, Xu NG*, et al.* Meta-analysis of the curative effect of acupuncture on post-stroke depression. Journal of traditional Chinese medicine, 2012,32(1): 6-11.
4. Zhang JB, Ren L, Sun Y. Meta-analysis on acupuncture for treatment of depression in patients of poststroke. Chinese Acupuncture & Moxibustion, 2009,29(7): 599-602.
5. Zhang ZJ, Chen HY, Yip KC*, et al.* The effectiveness and safety of acupuncture therapy in depressive disorders: systematic review and meta-analysis. Journal of Affective Disorders, 2010,124(1-2): 9-21.
6. Zhang XY, Li YX, Liu DL*, et al.* The effectiveness of acupuncture therapy in patients with post-stroke depression: An updated meta-analysis of randomized controlled trials. Medicine, 2019,98(22): e15894.
7. Zhan J, Tan F, Cheng NF*, et al.* Efficacy of Electroacupuncture and Antidepressants for Post-Stroke Depression: A Systematic Review of Randomized Controlled Trials. Chinese Archives of Traditional Chinese Medicine, 2016,34(10): 2379-2383.
8. Li XH, Chen JQ, Wang HT*, et al.* Comparison between Effects of Electroacupuncture and Antidepressants for Post Stroke Depression: A Systematic Review. Chinese General Practice, 2012,15(7): 802-806.
9. Zhou X, Ren L, Gao YY*, et al.* Meta-Analysis of Effect of Acupuncture and Antidepressants on Depression in Patients with Post-Stroke Depression. Chinese Archives of Traditional Chinese Medicine, 2018,36(12): 2875-2879.
10. Xu RX, Chen J. Meta-Analysis and Sequential Analysis of Depression in Acupuncture Treatment of Post-stroke Depression Patients. Chinese Journal of Gerontology, 2019,39(23): 5722-5726.
11. Zhang W, Sun JH, Gao Y*, et al.* System review on treating post-stroke depression with acupuncture. World Journal of Acupuncture-Moxibustion, 2014,24(2): 52-59.
12. Cx B, Lin SH, Jiang L. Comparison of different treatment methods for post-stroke depression. *China Practical Medicine* 2010; 5:71–3.
13. Chen SH. Clinical observation of electroacupuncture in the treatment of post-stroke depression. Chinese Journal of Primary Medicine and Pharmacy 2012, 19:3094–5.
14. Chen XZ, Wang J, Wang JH, et al. Clinical research into treatment of PSD with electrical needle for invigorating brain and easing mental stress. Liaoning journal of TCM 2005, 32:464–5.
15. Chu YJ, Wang CY, Zhang H. A clinical observation of acupuncture treatment of 72 cases with post-stroke depression. Chinese Journal of Gerontology 200, 27:1720–1.
16. Dong JP, Sun WY, Wang S, et al. Clinical observation on head point-through-point electroacupuncture for treatment of poststroke depression. Zhongguo Zhen Jiu 2007, 27:241–4.
17. Guo AS, Ah L, Chen X, et al. Effects of electric-acupuncture and fluoxetine on depression and neurological function of post-stroke depression patients. Shandong Medical Journal 2011, 51:9–11.
18. Hong ZM, Wang ZL, Zhang SQ, et al. Observation on effect of “Xingshen Jieyu” acupuncture method in treating post stroke depression. J Zhejiang Chin Med University 2015, 39:621–4.
19. Huang SL, Wei Y, Zhang ZR. Combination of electroacupuncture on “shen-wu-xing points” and western medicine for post-stroke depression: a randomized controlled trial. Shanghai J Trad Chin Med 2014, 48:33–6.
20. Kang WG, Yang BG. Electro-acupuncture therapy for the post-stroke depression. J Clini Acupuncture and Moxibustion 2014,3 0:35–7.
21. Jb L, Xm Y, Cheng RD, et al. Effect of electroacupuncture on regional cerebral blood flow in patients with poststroke depression. Chin J Rehabilitation Theory and Prac 2015, 21:192–5.
22. Xy Li, Shi GC, Dong QJ. Electroacupuncture on “tou-sanshen” and “si-guan-xue” for the treatment of 70 patients with post-stroke depression. Heilongjiang J Trad Chin Med 2013, 42:31–2.
23. Long HW, Tan PZ, Feng JH, et al. Clinical observation of electroacupuncture in the treatment of post-stroke depression. Journal of Clinical Psychiatry 2004, 14:173–4.
24. Peng HY, Js Y, Xj H, et al. Electroacupuncture versus fluoxetine capsule for post-stroke depression: a randomized controlled trial. Jilin J Trad Chin Med 2011, 30:990–1.
25. Wang YW, Wang CM, Sun YY. Clinical observation of electroacupuncture with different current frequencies for post-stroke depression. Shanghai J Acupu and Moxibustion 2015, 34:822–4.
26. Gq X, Miao GY. Clinical observation of electroacupuncture for elderly post-stroke depression. Chinese Journal of Convalescent Medicine 2015, 24:384–6.
27. Zhou Y, Jin JH, Zhou GY. Treatment of 145 post-stroke depression patients with electric acupuncture. J Shaanxi University of Chin Med 2010, 33: 78–80.
28. Zhou ZM. Electroacupuncture versus medicine in the treatment of post-stroke depression: a randomized controlled trial. China Medical Herald 2007, 4:127–8.
29. Zhu GY, Xm Y, Jb L, et al. Electroacupuncture on “kaisi-guan” versus fluoxetine in the treatment of post-stroke depression: a randomized controlled trial. Zhejiang J Integrated Trad Chin and Western Med 2012, 22:865–7.
30. Guo RY, Su L, Liu LA, et al. Effects of Linggui Bafa on the therapeutic effect and quality of life in patients of post-stroke depression. Zhongguo Zhen Jiu. 2009, 29(10):785-790.
31. Chi H. Clinical observation on post-stroke depression treated by “Tiao Qi Tong Du” acupuncture therapy. Haerbin: Helongjiang University of Chinese Medicine, China. 2011.
32. He XJ, Lai XS, Tan JL, et al. Clinical study on acupuncture in the treatment of post-stroke depression on with the method of activating the Du meridian and clearing the mind. Shijie Zhenjiu Zazhi. 2006, 16(3):8-12, 27.
33. Zheng MF, Zhang YS, Yuan CL, et al. Effect of “regulating Du channel and Ren channel” acupuncture method on initial post-stroke depression. Fu Jian Zhong Yi Xue Yuan Xue Bao. 2010, 1(1):16-18.
34. Yuan P, Zhang YJ, Dong GR. The clinical study for the treatment of post-stroke depression with scalp penetration acupuncture. Zhen Jiu Lin Chuang Za Zhi. 2006, 22(11):3-4.
35. Yang M. Chendu: The clinical study of acupoint application on the treatment of post-stroke depression. Chengdu: Chengdu University of Chinese Medicine, China. 2003.
36. Ding Z, Yu XG. Clinical study on treatment of post-stroke depression with acupuncture of du meridian as main therapy. Bei Jing Zhong Yi Yao Da Xue Xue Bao. 2003, 10(3):31-32.
37. Zhou CX, Cui X, Hu YS, et al. Effect of combined use of acupuncture and medicine on the activities of daily living in the treatment of post-stroke depression. Shanghai Zhenjiu Zazhi. 2012, 31(4):228-230.
38. Li HJ, Zhong BL, Fan YP, et al. Acupuncture for post-stroke depression: a randomized controlled trial. Zhongguo Zhen Jiu. 2011; 31(1):3-6.
39. Li CP. The clinical study on acupuncture of “Shu Gan Li Qi” in treatment of the post-stroke depression. Haerbin: Heilongjiang University of Chinese Medicine, China. 2009.
40. Liu YR. Qantizational observation on acupuncture and point-injection treatment of baihui acupoint for post cerebral infarction depression. Guangzhou: Guangzhou University of Chinese Medicine, China. 2008.
41. Ma LY, Gong SF, Huo J. Clinical observation of Yu’s scalp acupuncture on poststroke depression. Zhen Jiu Lin Chuang Za Zhi. 2012, 28(5):66-68.
42. Sun XW, Zou W, Li HT, et al. The Clinical study of ‘Tiao Shen Jie Yu’ acupuncture treatment on post-stroke depression. The elderly medical academic conference in conjunction with the world in the third session of Chinese medicine, traditional Chinese and Western Medicine. 2010.
43. Sun YZ, Jia SY. Clinical study on Yu’s cluster needling at scalp acupoints for post-stroke depression. Shang Hai Zhen Jiu Za Zhi. 2012, 31(8):564-556.
44. Wang LJ. Clinical research of treatment of the “Gan Qi Yu Jie” post-stroke depression by acupuncture. Shenyang: Liaoning Zhongyiyao Daxue. 2010.
45. Zhu SS. The impact of brain-reinforcing and mind-regulating acupuncture on the life of post-stroke depression patient. Nanjing: Nanjing University of Chinese Medicine, China. 2008.
46. He FR. Effect on acupuncture therapy of “Tong Tiao Du Ren” in initial post-stroke depression. Xiamen: Fujian University of Traditional Chinese Medicine. 2009.
47. Zhang CZ. Observations on curative effect of acupuncture for restoring consciousness and inducing resuscitation on 45 PSD patients. New TCM 2004, 36: 50-51.
48. Sun JH, Zhu SS, Pei LX. Clinical observations on treatment of 30 PSD patients with acupuncture for invigorating brain and regulating mind. Jiangsu TCM 2004, 41:46-47.
49. Dai W. Observations on curative effect of electrical needle on PSD. Hubei Journal of TCM 2009, 9: 22-23.
50. He XJ, Lai XS, Clinical research into treatment of PSD with acupuncture for clearing Du channel and restoring consciousness. In: Society of Deficiency Syndrome and Senile Disease, Chinese Association of Integrative Medicine, editor. Proceedings of the 9th national conference on treatment of deficiency syndrome and senile disease with integrative medicine, 2007 Aug 15-17; Changchun, Jilin, China. Beijing: Chinese Association of Integrative Medicine,161-164.
51. Jiang F, Li CD. Clinical observations on curative effect of acupuncture at Wuzhi point on PSD. Clinical Journal of Acupuncture 2006, 22: 25-26.
52. Li Y, Liu R, Wang Y, Xu WY. Treatment of 36 PSD patients with acupuncture for regulating mind and Qi. Clinical Journal of Acupuncture 2009, 25: 22-23.
53. Wang P, Ji QM, Huo XL. Clinical observations on treatment of PSD mainly with scalp acupuncture. Shanghai Journal of Acupuncture 2004, 23: 15-16.
54. Zhang XD, Tan JX. Treatment of different types of PSD with point injection. Guangming TCM 2009, 24: 1335-1336.
55. Wei GW. Clinical observations on treatment of 30 PSD patients with acupuncture. Liaoning Journal of TCM 2008, 35: 1563-1564.
56. Wang HJ. Clinical observations on treatment of PSD with acupuncture. China Acupuncture 2003, 23: 442-443.
57. Zhuang ZQ, Wang CR. Treatment of PSD with "intelligent 3 needles" plus point massage. China Acupuncture 2004, 24: 800-803.
58. Zhang Y. Research into influence of acupuncture on PSD and recovery of nervous function. Modern Journal of Combination of TCM and Western Medicine 2004,13: 864-865.
59. Jiang Z Y, Gao L, Li C D, et al. Clinical study on the treatment of post-stroke depression with electroacupuncture of “niwan bazhen”points [J]. World J Acup moxi ,2007,17(1) :11 - 16.
60. Cui H. Electric acupuncture treat 30 cases of melancholia after Apoplexy [J]. Journal of Zhejiang Chinese Medical University, 2005 ,29 (2) :65 - 66.
61. Nie B, Nie T. Clinical study on electroacupuncture treatment of post-apoplectic depression [J]. Shanghai Journal of Acupuncture and Moxibustion, 2006 ,25 (9) :6 - 8.
62. Xing XL, Jiang ZH, Nie YB, Xu ZX. Clinical Study on the Treatment of Post-apoplectic Melancholia with the Tongue Needle and Chinese Herbal medicine [J]. Lishizhen Medicine and Materia Medica Research, 2005 ,16 (12) :1268 - 1269.
63. Wang Y, Zhao, ZF., Fu L, et al. An evaluation on the efficacy of acupuncture treatment of insomnia and depression in patients with stroke. Zhong Guo Zhen Jiu 2004, 24, 603–606.
64. Li XJ. Clinical observations on the treatment of post-apoplectic melancholia with scalp points [J]. Shanghai Journal of Acupuncture and Moxibustion, 2004 ,23 (10) :13 - 14.
65. Liu SK, Zhao XM, Xi ZM. Incidence rate and acupuncture-moxibustion treatment of post-stroke depression [J]. Chinese Acupuncture & Moxibustion, 2006 ,26 (7) :472 - 474.
66. Sun L, Shen PF. Observation on the therapeutic effect of Xingnao Kaiqiao acupuncture on 180 cases of post-stroke depression [J]. Journal of New Chinese Medicine, 2007 ,39 (10) :27 - 28.
67. Wang CX. Observation on Therapeutic Effect of Xingnao Tongdu Acupuncture Treatment on post-stroke Depression [J]. Liaoning Journal of Traditional Chinese Medicine, 2006 ,33 (5) :601 - 602.
68. Gu W. Clinical observation on acupuncture treatment of post-stroke depression [J]. Journal of Sichuan of Traditional Chinese Medicine, 2005 ,23 (10) :102 - 103.
69. Yang DR, Yan WQ. Observation on therapeutic effect of acupuncture on post-stroke depression [J]. Chinese Journal of Information on Traditional Chinese Medicine, 2007 ,14 (7) :75 - 76.
70. Li KS, Zeng XQ. Clinical Observation on Acupuncture and Cupping in Treating Post-stroke Depression [J]. Journal of Clinical Acupuncture and Moxibustion, 2007 ,23 (5) :36 - 37.
71. Zhang YK, Jiang YX. Observations on the efficacy of combined acupuncture and medicine for treating 30 post-stroke depression patients [J]. Shanghai Journal of Acupuncture and Moxibustion, 2007 ,26 (4) :5 - 6.
72. Cheng Y, Zhao JJ, 2007. A randomized controlled trial of abdominal acupuncture in patients with post-stroke depression. Zhong Hua Zhong Yi Yao Xue Kan 25, 1888–1890.
73. Huang DH, Wang CY, Huang JH, et al. 2004a. Acupuncture on Baihui combination acupoint injection with herbal extractive liquid in the treatment of post-stroke depression. Zhong Guo Lin Chuang Kang Fu 8, 6132–6133.
74. Peng HY., Tan JL, 2007. A Clinical Trial of Temporal Tri-Needle Acupuncture as a Principal Therapy for the Treatment of Post-Stroke Depression. Degree Thesis of Guangzhou University of TCM 14–20.
75. Tang JX, Guan NJ, Li L, et al. 2003. The efficacy of electroacupuncture treatment of post-stroke depression and effects on the quality of life for patients. Shanghai Zhen Jiu Za Zhi 22, 12–14.
76. Yin CP, 2004. A clinical observation of antidepressant and acupuncture combination treatment of post-stroke depression in 100 cases. Xin Zhong Yi 36, 24–25.
77. Zhai TJ, Luo EL, Guo SJ, 2004. Antidepressant effects of acupuncture on poststroke depression and rehabilitation. Zhen Jiu Lin Chuang Za Zhi 20, 5–7.
78. Zhao H, Zhao WL. 2007. A Clinical Study of Acupuncture With Baihui Acupoint in Patients With Post-Stroke Depression. Zhong Hua Zhong Yi Yao Xue Kan 28, 199–200.
79. Liu ZL, Lao JX, Pan QJ. Clinical observations on warm needling of back shu points for intervention in post-stroke depression. Shanghai J Acupunct Moxibustion 2013; 32:255–7.
80. Cao XJ, Song WX, Clinical observation on acupuncture therapy of resolving phlegm and depression and activating blood and channels for post stroke depression. Clin J Chin Med 2014; 6:7–9.
81. Liao BD. Observation of curative effect by acupuncture and moxibustion therapy for post-stroke depression. Chin J Modern Drug Appl 2017; 11:122–4.
82. Chu LC, Cheng WP. Clinical analysis of acupuncture and moxibustion in treatment of acute post-stroke depression. Heilongjiang Sci 2017;8:164–5.
83. Yan CC, Su SY, Tang GK, et al. Clinical study on acupuncture and moxibustion in the treatment of post-stroke depression. Shanxi J Tradit Chin Med 2018;39:122–4.
84. Sun HD, Wang F. Therapeutic effect of electroacupuncture on poststroke depression. Chin J Clin Rational Drug Use 2015; 8:98–9.
85. Zhang JL, Wang F. Treatment of 34 cases of post-stroke depression with electroacupuncture at ear points [J]. Journal of External Therapy of Traditional Chinese Medicine, 2013, 1: 46－47．
86. Huang L, Sun ZR. Clinical observation of electroacupuncture in the treatment of post-stroke depression [J]. Journal of Clinical Acupuncture and Moxibustion, 2005, 5: 48 － 49．
87. Yao S. The clinical observation of electroacupuncture’s treatment of post-stroke depression on the acupoint of forehead [D]. Harbin: Heilongjiang University of Chinese Medicine, 2010.
88. Wang YW. High-frequency electroacupuncture for 40 cases of post-stroke depression [J]. Zhejiang Journal of Traditional Chinese Medicine, 2010, 45(1):62.
89. Chen MD. Clinical observation on scalp electroacupuncture for treatment of post-stroke depression [D]. Changsha: Hunan University of Chinese Medicine, 2009.
90. Long HW, Tan PZ, Feng JH, Li MZ. Clinical observation of electroacupuncture in the treatment of post-stroke depression [J]. Journal of Clinical Psychiatry, 2004, 14 ( 3) : 173-174
91. Xin LH. Clinical Study on the Treatment of Post-stroke Depression by Jiannao Anshen [D]. Changchun: Changchun University of Chinese Medicine,2008.
92. Chen QB. Effect evaluation of acupuncture combined with medicine intervention on post-stroke depression [J]. China Health Monthly: Academic Edition, 2011, 30 (2): 190.
93. Wang WH, Zhang QY. Clinical observation of acupuncture combined with Fluoxetine in treating post-stroke depression [J]. China's Naturopathy, 2011, 19(7): 50-51
94. Wu Y, Sun XH, Xing Y. Intervening effect of point-to-point acupuncture on post-stroke depression and its influence on plasma hydrocortisone levels [J]. Shanghai Journal of Acupuncture and Moxibustion, 2011, 30(3): 153-154．
95. Liu YH. Clinical observation of acupuncture combined with Xiaoyao San in the treatment of post-stroke depression [J]. National Medical Frontiers of China, 2012, 7(18): 36,42．
96. Shan, N., Curative observation of Liushen point moxibustion combined with emotional nursing treatment for treating depression after stroke [J]. Journal of Sichuan of Traditional Chinese Medicine, 2017, 35(2) : 180－182．
97. Kong XY, Study on acupuncture combined with back Shu points in patients with depression after stroke of depressive function [J]. Journal of Liaoning University of Traditional Chinese Medicine, 2015, 17(7): 191-193.
98. Zhang J, Wang W, Wu HH, Liu SJ, Wang YM. Clinical observation of 60 cases of cerebral apoplexy treated with acupuncture combined with electro acupuncture and electro acupuncture [J]. World Latest Medicine Information, 2015,15(94):52-53,59.
99. Zhu YG, Zhao Q, Bai XY, Su QL, Xu YD, Yang, J. Observation of clinical efficacy of scalp acupuncture with ear-point pressure in treating post-stroke depression [J]. Journal of Nanjing University of Traditional Chinese Medicine, 2014, 30(4): 323-325．
100. Yang SQ, Wang WH, Observations on the efficacy of scalp acupuncture plus medicine for post-stroke depression [J]. Shanghai Journal of Acupuncture and Moxibustion, 2013, 32(1): 9-11．
101. Duan JY, Ding BY, Zong L. Clinical Observation of Acupuncture at the Thirteen Ghost Acupoints for Post-stroke Anxiety and Depression [J]. Shanghai Journal of Acupuncture and Moxibustion, 2014, 33(6): 536-538．
102. Wang GC, Xiao W, Zhang XB, Liang FJ, Wang Z, Kong HB, Guo XL, Hu HX, Zhang CQ. Study of Tongduzhiyu acupuncture in treating patients of PSD by influencing 5-HT, NE and BDNF in serum [J]. Journal of Gansu University of Chinese Medicine, 2015, 32(2): 58-62.
103. Wang F, Pan W, Li YF. Therapeutic observation of acupuncture plus auricular point electroacupuncture for post-stroke depression and its effect on quality of life [J]. Shanghai Journal of Acupuncture and Moxibustion, 2016, 35(9): 1033-1035．
104. Nie RR, Huang CH. Post-stroke depression treated with acupuncture and moxibustion: an evaluation of therapeutic effect and safety [J]. Chinese Acupuncture & Moxibustion, 2013, 33(6):490-494.
105. Lu BB, Chen JJ. Clinical observation on acupuncture and herb medicine combined with psychotherapy in treating post-stroke depression [J]. Guide of China Medicine, 2013, 11(32): 198-199．
106. Chen HM, Gao M, Yu T. Clinical observation on Jiawei Sini powder combined with abdominal acupuncture in treating post-stroke depression [J]. Stroke and Nervous Diseases, 2016. 23(5): 347-349．
107. Chen WM, Feng WX, Pan HS. 29 case of post-stroke depression treated by Xingnao Kaiqiao acupuncture combined with sertraline [J]. Journal of External Therapy of Traditional Chinese Medicine, 2018; 27(2): 20-1.
108. Feng GF, Li JY, Wen QF. Effect of acupuncture at back Shu Points of five zang organs on post-stroke depression [J]. Shenzhen Journal of Integrated Traditional Chinese and Western Medicine, 2018; 28(14): 56-8.
109. Guo XD, Wang ML, Zhao XW, Hou DM, Qi YM. Effect of rehabilitation training combined with acupuncture intervention on post-stroke depression and activities of daily living [J]. Chinese Journal of Physical Medicine and Rehabilitation, 2012; 34(9): 711-2.
110. Jiao DY, Deng HP, Gu HY, Wang L, Zhang RC. Clinical study on treatment of poststroke depression by acupuncture combined with conventional therapy of western medicine [J]. International Journal of Traditional Chinese Medicine, 2017; 39(12): 1065-8.
111. Jing SQ, Chen H, Zhang XY, Huang ZJ. Effect of acupuncture on neurological rehabilitation of post-stroke depression. [J] Journal of Modern Medicine & Health, 2018, 34(16): 2559-61.
112. Li JM. Effect of acupuncture combined with rehabilitation training on cognitive function of patients with post-stroke depression [J]. Chinese Journal of Practical Nervous Diseases, 2018, 21(16): 1820-6.
113. Liu JZ, An QY. Acupuncture combined with fluoxetine in the treatment of 52 elderly patients with post-stroke depression [J]. Henan Traditional Chinese Medicine, 2014,34(9): 1700-1.
114. Sun PY, Chu HR, Li PF, Wang T, Pu F, Wu J, Liu X, Luo CM, Liu Y. The effect of the acupuncture intervention of dredging Governor Vessel and regulating mentality for the medication treatment of post-stroke depression [J]. Chinese Acupuncture & Moxibustion, 2015,35(8): 753-7.
115. Sun XD, Yang N, Che WS. Curative Effects of Acupuncture on Post-stroke Depression and its Effect son Neurotrophic Status and Monoamine Neurotransmitters [J]. World Chinese Medicine, 2018;13(9): 2285-7, 2291.
116. Sun YT, Bao YH, Wang SL, Chu JM, Li LT. Efficacy on post-stroke depression treated with acupuncture at the acupoints based on ziwuliuzhu and Prozac [J]. Chinese Acupuncture & Moxibustion, 2015;35(2): 119-22.
117. Ye QJ. Clinical analysis of 43 cases of post-stroke depression treated by tiaoshengshugan acupuncture combined with fluoxetine [J]. Chinese Journal of Ethnomedicine and Ethnopharmacy, 2013,22(23): 62.
118. Tong X, Liu DD, Wei Y, Kou JY, Yang TS, Qiao LD. Intervening effect of mind-regulating and liver-soothing acupuncture therapy on post-stroke depression [J]. China Medicine and Pharmacy, 2012,2(3): 127-8.
119. Wang YB, Shi HY, Chen HS. Clinical observation of acupuncture combined with Prozac in the treatment of post-stroke depression [J]. Yiyao Qianyan, 2018,8(15): 331.
120. Wu XM, Zhang B. Clinical observation on 40 cases of post-stroke depression treated by acupuncture combined with Prozac [J]. Chinese Journal of Difficult and Complicated Cases, 2008,7(6): 357-8.
121. Xia JH, Xu CE, Xia WG, Zheng CJ. Clinical Observation of Acupuncture plus Rehabilitation Training for Post-stroke Depression [J]. Shanghai Journal of Acupuncture and Moxibustion, 2015,(8): 724-7.
122. Yao ZY, Yang JR, Yan CL. The clinical effect of Xiaoyao powder combined with acupuncture for post-stroke depression [J]. Gansu Medical Journal, 2018,37(1): 67-8.
123. Yu WY. Acupuncture combined with fluoxetine hydrochloride capsule in the treatment of 87 cases of post-stroke depression [J]. Hebei Journal of Traditional Chinese Medicine, 2010,32(4): 567-8.
124. Gao YY. Effects of electroacupuncture on serum levels of RBP-4 and Hp in patients with post-stroke depression [D]. Qingdao: Qingdao University: 2013.
125. Nie RR. Clinical studies of treating PSD with acupuncture or regulating liver and strengthening the foundation (Chin). Guangzhou: Guangzhou University of Chinese Medicine; 2012.
126. Wang LJ. Clinical studies of treating stagnation-type PSD with acupuncture (Chin). Shenyang: Liaoning University of Traditional Chinese Medicine; 2011.
127. Fu L. Clinical observation of treating post-stroke depression by acupuncturing Sìshéncōng (EXHN 1) and back-shu point (Chin). Changsha: Hunan University of Chinese Medicine; 2013.
128. Nie RR, Huang CH, Fu WB. Therapeutic observation of treating post-stroke depression by differentiation of spleen and stomach. Chin Acup-Mox (Chin) 2011; 31(4): 325-326.
129. Sun PY, Chu HR, Li PF, Wang Y, Li F, Xia JG, et al. Treating post-stroke depression with regulating governor vessel and the mind: randomized controlled trial. Chin Acup-Mox (Chin) 2013; 33(1): 3-7.
130. He, J., Shen, P.F., 2007. A clinical study evaluating the efficacy of acupuncture post-stroke depression. Zhen Ci Yan Jiu 32, 58–61.

| **Table S4** Excluded trials and reasons for exclusion | |
| --- | --- |
| Excluded Trials | Reason for exclusion |
| Wang 2006 ^1^ | This is a non- randomized trial (The randomization method is in the order of admission.) |
| Wei 2008 ^2^ | This is a non- randomized trial (The randomization method is in the order of visit.) |
| Wang 2003 ^3^ | This is a non- randomized trial (The randomization method is in the order of visit.) |
| He 2007 ^4^ | This is a non- randomized trial (The randomization method is in the order of visit.) |
| Niu 2015 ^5^ | This is a non- randomized trial (The randomization method is in the order of admission.) |
| Zhao 2007 ^6^ | This trial didn’t put forward the randomization method. |
| Jiao 2017 ^7^ | This trial didn’t report appropriate data on HAMD scale. |
| Dong 2007 ^8^ | This study is a three-arm trial, the acupuncture group was divided into a point-through-point EA group and a non-point-through-point group. |
| Man 2014 ^9^ | This trial included body electroacupuncture both in acupuncture and control group. |
| Li 2017 ^10^ | Both treatment and control group patients received acupuncture treatment. |
| Huang fu 2018 ^11^ | This study is a three-arm trial, the acupuncture group was divided into a traditional acupuncture group and “Guaisanzhen” group. |
| Shen 2010 ^12^ | Both treatment and control group patients received acupuncture treatment. |
| Ma 2015 ^13^ | Both treatment and control group patients received acupuncture treatment. |
| Zhuang 2004 ^14^ | The treatment group was acupuncture combined with acupressure. |
| Nie 2011 ^15^ | The treatment group was acupuncture combined with grain moxibustion. |
| Xing 2005 ^16^ | The treatment group was acupuncture combined with herbs. |
| Yao 2017 ^17^ | The treatment group was acupuncture combined with psychological intervention, but the control group only contained drugs. |
| Zhang 2017 ^18^ | The treatment group received acupuncture plus auricular point sticking. |
| Zhang L 2017 ^19^ | The treatment group received acupuncture plus auricular point sticking. |
| Wang 2015 ^20^ | The treatment group was acupuncture combined with health education, but the control group only contained drugs. |
| Zhou 2015 ^21^ | The treatment group was acupuncture combined with moxibustion. |
| Nie 2013 ^22^ | The treatment group was acupuncture combined with grain moxibustion. |
| Zhao 2019 ^23^ | This is a non- randomized trial (The randomization method is in the order of admission.) |

**References**

1. Wang CX. Observation on Therapeutic Effect of Xingnao Tongdu Acupuncture Treatment on post-stroke Depression [J]. Liaoning Journal of Traditional Chinese Medicine, 2006 ,33 (5) :601 - 602.
2. Wei GW. Clinical observations on treatment of 30 PSD patients with acupuncture. Liaoning Journal of TCM 2008; 35: 1563-1564.
3. Wang HJ. Clinical observations on treatment of PSD with acupuncture. China Acupuncture 2003; 23: 442-443.
4. He J, Shen PF, 2007. A clinical study evaluating the efficacy of acupuncture post-stroke depression. Zhen Ci Yan Jiu 32, 58–61.
5. Niu YL, Liu CM, Wang XD, Wang LN, Feng XD. Effect of Acupuncture Combined with Moxibustion on Heart Rate Variability in Patients with Post-stroke depression [J]. Chinese Journal of Rehabilitation Theory and Practice, 2015,21(02):196-198.
6. Zhao H, Zhao WL, 2007. A Clinical Study of Acupuncture With Baihui Acupoint in Patients With Post-Stroke Depression. Zhong Hua Zhong Yi Yao Xue Kan 28, 199–200.
7. Jiao DY, Deng HP, Gu HY, Wang L, Zhang RC. Clinical study on treatment of poststroke depression by acupuncture combined with conventional therapy of western medicine [J]. International Journal of Traditional Chinese Medicine, 2017; 39(12): 1065-8.
8. Dong JP, Sun WY, Wang S, et al. Clinical observation on head point-through-point electroacupuncture for treatment of poststroke depression. Zhongguo Zhen Jiu 2007; 27:241–4.
9. Man S C , Hung B H B , Roger M K Ng…. A pilot controlled trial of a combination of dense cranial electroacupuncture stimulation and body acupuncture for post-stroke depression[J]. BMC Complementary and Alternative Medicine, 2014, 14(1):255.
10. Li, Menghan, Zhang,et.al. Effect of Tiaoshen Kaiqiao acupuncture in the treatment of ischemic post-stroke depression: a randomized controlled trial[J]. Journal of Traditional Chinese Medicine, 2017.
11. Huangfu L, Yang QY, Liu F*, et al.* Effect of Dong's Extraordinary Acupoints “Guaisanzhen” for Post-stroke Depression [J]. Chinese Archives of Traditional Chinese Medicine, 2018(10):2325-2328.
12. Shen Q, Qiu JZ, Huang Y*, et al.* A clinical study on treating post-stroke depression by head matrix acupuncture [J]. Liaoning Journal of Traditional Chinese Medicine, 2010, 37(4):718-720.
13. Ma QP, Pang Y, Ji S. Effect analysis of kidney- reinforcing and du meridian- regulating acupuncture on post- stroke depression and its influence on life quality [J]. Liaoning Journal of Traditional Chinese Medicine, 2015(06):173-176.
14. Zhuang ZQ, Wang CR. Treatment of PSD with "intelligent 3 needles" plus point massage. China Acupuncture 2004; 24: 800-803.
15. Nie RR, Huang CH, Fu WB. Therapeutic observation of treating post-stroke depression by differentiation of spleen and stomach. Chin Acup-Mox (Chin) 2011; 31(4): 325-326.
16. Xin XL, Jiang ZH, Nie YB*, et al.* Clinical study on the treatment of post-apoplectic melancholia with the Tongue needle and Chinese herbal medicine [J]. Lishizhen Medicine and Materia Medica Research, 2005 ,16(12): 1268–1269.
17. Yao LJ, Liu ZQ, Zhou J*, et al.* The clinical study of Tongdu Tiaoshen needling combined with cognitive therapy for post-stroke depression patients [J]. Modernization of Traditional Chinese Medicine and Materia Medica-World Science and Technology, 2017, 19(8):1319-1323.
18. Zhang, Lin, Zhong, Yan, Quan,等. Effect of combining acupuncture and auricular point sticking on heart rate variability in patients with post-stroke depression[J]. Journal of Acupuncture & Tuina Science, 2017, 15(6):392-397.
19. Zhang L, Zhong Y, Quan SL*, et al.* Acupuncture combined with auricular point sticking therapy for post stroke depression: a randomized controlled trial [J]. Chinese Acupuncture & Moxibustion, 2017, 37(6):581-585.
20. Wang DY, Zhou WJ, Ding J*, et al.* Effect of acupuncture and health education on functional rehabilitation and serum SCD40L of depressed patients with cerebral infarction [J]. Chinese Journal of Critical Care Medicine, 2015,35(7): 83-84.
21. Zhou XF, Li Y, Zhou ZH*, et al.* Clinical observation of acupuncture in patients with depression and its impact on serum 5-HT [J]. Chinese Acupuncture & Moxibustion, 2015, 35(02):123-126.
22. Nie RR, Huang CH. Post-stroke depression treated with acupuncture and moxibustion: an evaluation of therapeutic effect and safety [J]. Chinese Acupuncture & Moxibustion, 2013, 33(6):490-494.
23. Zhao JX, Li F. Effect of acupuncture on post-stroke depression. 2019, 12(7):9097‐9103.

| **Table S5** Results of sensitivity analyses excluding the listed trials | | | | | | |
| --- | --- | --- | --- | --- | --- | --- |
| **Removed trials** | **Reasons of deletion** | **No of studies** | **No of participants** | **Relative risk (95% CI)** | ***I^2^* – value (%)** | ***P* - value** |
| **Acupuncture/ EA + conventional treatment vs. conventional treatment** | | | | | | |
| **Sun Y 2015 ^1^** | | | | | | |
| Before sensitivity analysis | Moderate and severe depression degree | 3 | 252 | MD, -5.08 (-6.48, -3.67) | 0 | 0.00001 |
| After sensitivity analysis | Moderate and severe depression degree | 2 | 190 | MD, -5.28 (-7.02, -3.54) | 0 | 0.00001 |
| **HAMD scale - 17 item (Acupuncture/ EA (+placebo) vs. antidepressants (+sham acupuncture))** | | | | | | |
| **Li 2011 ^2^, Qian 2015 ^3^** | | | | | | |
| Before sensitivity analysis | Combined with placebo | 5 | 257 | MD, -0.43 (-1.61, 0.75) | 51 | 0.47 |
| After sensitivity analysis | Combined with placebo | 3 | 148 | MD, -1.04 (-1.85, -0.22) | 0 | 0.01 |
| **HAMD scale (Acupuncture/ EA (+placebo) vs. antidepressants (+sham acupuncture))** | | | | | | |
| **Zhou 2016 ^4^** | | | | | | |
| Before sensitivity analysis | Low quality | 4 | 494 | MD, -1.55 (-4.36, 1.26) | 95 | 0.28 |
| After sensitivity analysis | Low quality | 3 | 378 | MD, -0.19 (-1.00, 0.62) | 0 | 0.64 |
| **Liu 2006 ^5^, Chu 2007 ^6^** | | | | | | |
| Before sensitivity analysis | Contained mild depression degree | 4 | 494 | MD, -1.55 (-4.36, 1.26) | 95 | 0.28 |
| After sensitivity analysis | Contained mild depression degree | 2 | 176 | MD, -2.62 (-6.93, 1.69) | 97 | 0.23 |
| **Adverse events (Acupuncture/ EA (+placebo) vs. antidepressants (+sham acupuncture))** | | | | | | |
| **Li 2011 ^2^** | | | | | | |
| Before sensitivity analysis | Combined with placebo | 5 | 296 | RR, 0.16 (0.07, 0.39) | 35 | <0.0001 |
| After sensitivity analysis | Combined with placebo | 4 | 253 | RR, 0.08 (0.02, 0.29) | 0 | 0.0001 |

**References**

1. Sun YT, Bao YH, Wang SL*, et al.* Efficacy on post-stroke depression treated with acupuncture at the acupoints based on ziwuliuzhu and Prozac [J]. Chinese Acupuncture & Moxibustion, 2015,35(2): 119-122.
2. Li HJ, Zhong BL, Fan YP*, et al.* Acupuncture for post-stroke depression: a randomized controlled trial [J]. Chinese Acupuncture & Moxibustion, 2011,31(01):3-6.
3. Traditional Chinese Acupuncture for Poststroke Depression: A Single-Blind Double-Simulated Randomized Controlled Trial[J]. Journal of Alternative & Complementary Medicine, 2015, 21(12):acm.2015.0084.
4. Zhou MY, Wu LX. Clinical Effectiveness of Acupuncture in Patients with Depression and Observation on Safety Evaluation after stroke [J]. Chinese Archives of Traditional Chinese Medicine, 2016, 34(2):502-504.
5. Liu SK, Zhou XM, Xi ZM. Incidence rate and acupuncture-moxibustion treatment of post-stroke depression [J].2006 ,26(7):472 - 474.
6. Chu YJ, Wang CY, Zhang H. A clinical observation of acupuncture treatment of 72 cases with post-stroke depression. Chinese Journal of Gerontology 2007; 27:1720–1.

| **Table S6** The acupoints and their frequency of use in the included studies | |
| --- | --- |
| Acupoints | Frequency |
| PC6 | 9 |
| SJ5 | 1 |
| SP4 | 1 |
| GB41 | 1 |
| SI3 | 1 |
| BL62 | 2 |
| LU7 | 1 |
| KI6 | 2 |
| GV20 | 11 |
| GV29 | 3 |
| LR3 | 8 |
| HT7 | 7 |
| BL15 | 2 |
| SP6 | 6 |
| KI3 | 4 |
| ST36 | 5 |
| EX-HN1 | 7 |
| ST40 | 2 |
| LI4 | 3 |
| ST16 | 1 |
| GV24 | 5 |
| GV16 | 2 |
| GV26 | 4 |
| GV14 | 2 |
| GV11 | 2 |
| ST10 | 1 |
| EX-HN3 | 2 |
| DU24 | 1 |
| DU16 | 1 |
| GV15 | 1 |
| BL18 | 1 |
| BL23 | 1 |
| CV17 | 1 |
| DU26 | 1 |

| **Table S7** The treatment time and frequency in the included studies | |
| --- | --- |
| Treatment time | Frequency |
| 30min | 12 |
| 40min | 4 |
| 20min | 2 |
| 15min | 1 |

| **Table S8** The treatment time and frequency in the included studies | |
| --- | --- |
| Duration | Frequency |
| 30d | 3 |
| 6m | 3 |
| 4w | 4 |
| 28d | 1 |
| 60d | 1 |
| 6w | 3 |
